# TopFD: A Proteoform Feature Detection Tool for Top–Down Proteomics

**DOI:** 10.1021/acs.analchem.2c05244

**Published:** 2023-05-17

**Authors:** Abdul Rehman Basharat, Yong Zang, Liangliang Sun, Xiaowen Liu

**Affiliations:** † Department of BioHealth Informatics, School of Informatics and Computing, Indiana University-Purdue University Indianapolis, Indianapolis, Indiana 46202, United States; ‡ Department of Biostatistics and Health Data Sciences, Indiana University School of Medicine, Indianapolis, Indiana 46202, United States; § Department of Chemistry, Michigan State University, East Lansing, Michigan 48824, United States; ∥ Deming Department of Medicine, Tulane University School of Medicine, New Orleans, Louisiana 70112, United States

## Abstract

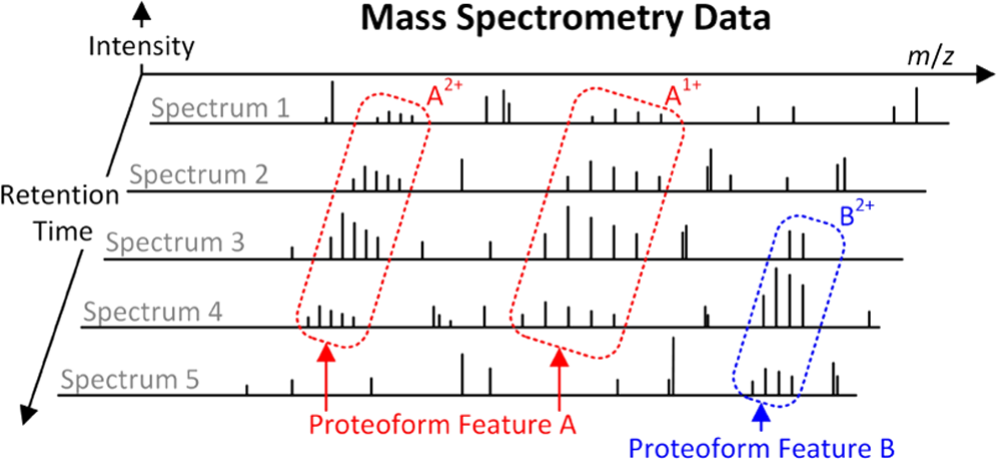

Top-down liquid chromatography-mass spectrometry (LC-MS) analyzes intact proteoforms and generates mass spectra containing peaks of proteoforms with various isotopic compositions, charge states, and retention times. An essential step in top-down MS data analysis is proteoform feature detection, which aims to group these peaks into peak sets (features), each containing all peaks of a proteoform. Accurate protein feature detection enhances the accuracy in MS-based proteoform identification and quantification. Here, we present TopFD, a software tool for top-down MS feature detection that integrates algorithms for proteoform feature detection, feature boundary refinement, and machine learning models for proteoform feature evaluation. We performed extensive benchmarking of TopFD, ProMex, FlashDeconv, and Xtract using seven top-down MS data sets and demonstrated that TopFD outperforms other tools in feature accuracy, reproducibility, and feature abundance reproducibility.

## Introduction

Top-down mass spectrometry (MS) has attracted increasing attention owing to its unique capacity to analyze intact proteoforms and characterize proteoforms with multiple alterations, such as sequence mutations, splicing events, and post-translational modifications (PTMs).^1−3^ Advances in high-resolution and high-accuracy MS instruments significantly increased proteoform identifications and amino acid sequence coverage in proteome-wide top-down proteomics analysis.^4^ Recent top-down MS studies identified more than 23 000 proteoforms from colorectal cancer cells^5^ and about 30 000 proteoforms from human blood and bone marrow cells.^6^ Top-down MS-based proteoform profiling has successfully identified differentially expressed proteoforms associated with diseases.^7−10^

**Figure 1:**
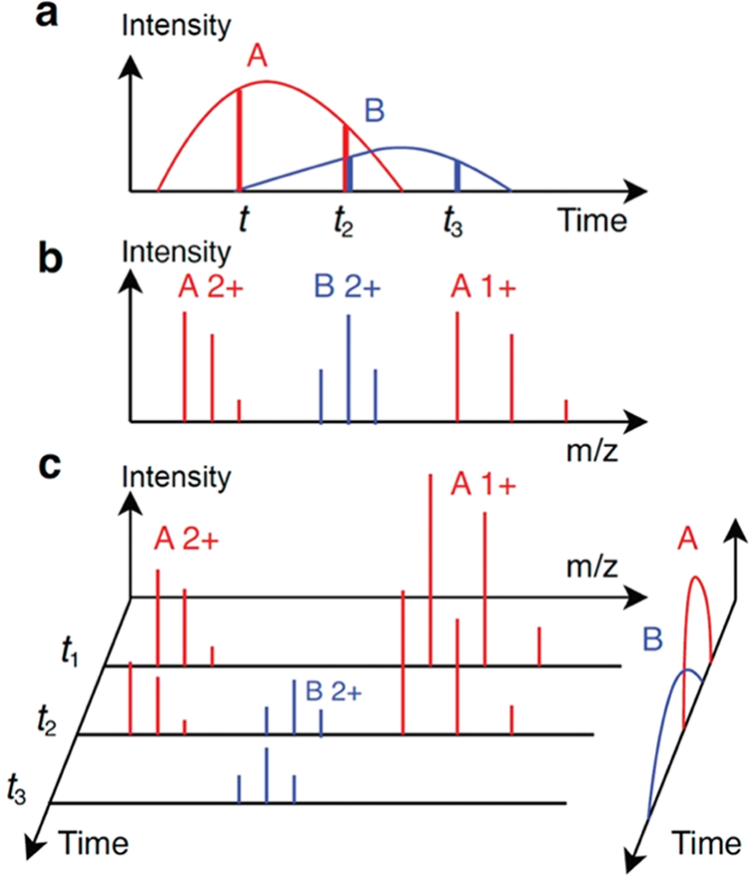
Illustration of two proteoform features in an LC-MS map. (a) Elution profiles (curves) of two proteoforms *A* and *B*. At time points *t*_1_, *t*_2_, and *t*_3_, three MS1 spectra are generated. The abundances of *A* have a ratio of 4:3:0 (two red vertical lines), and the abundances of *B* have a ratio of 0:1:1 (two blue vertical lines). (b) Theoretical isotopic envelopes of proteoform *A* with charge states 1+ and 2+ and proteoform *B* with charge state 2+. (c) Three MS1 spectra are generated at time points *t*_1_, *t*_2_, and *t*_3_. The elution profiles on the right are the same as (a). The three spectra contain four experimental isotopic envelopes of *A* and two envelopes of *B*.

Proteoform feature detection is a fundamental computational problem in top-down MS-based proteoform quantification. In proteome-wide top-down MS analysis, proteoforms extracted from samples are first separated by liquid chromatography (LC) or other separation methods and then analyzed by tandem mass spectrometry (MS/MS). Each proteoform has an elution profile (Figure 1a), which depicts the abundance of the proteoform eluted over time in proteoform separation. A mass spectrum contains a list of peaks, each represented by its mass-to-charge ratio (*m*/*z*) and intensity. The isotopologues of a proteoform with the same charge state are detected as a group of isotopic peaks in a mass spectrum, called an isotopic envelope (Figure 1b). The peak intensities in an isotopic envelope follow a distribution determined by the isotopic frequencies of the atoms in the proteoform. A mass spectrum often contains multiple isotopic envelopes of a proteoform with different charge states (Figure 1b). Feature detection in top-down MS aims to identify all isotopic peaks of each proteoform over retention time (RT) and across charge states in a liquid chromatography-mass spectrometry (LC-MS) data file and reports its elution profile and total signal intensity (Figure 1c).

**Figure 2:**
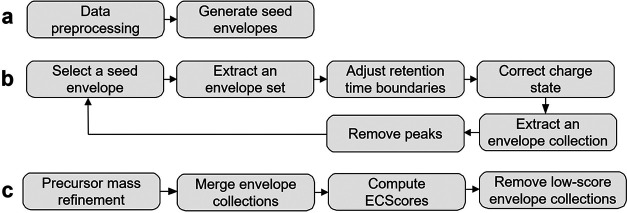
Overview of the pipeline for proteoform feature identification in TopFD. (a) Preprocessing. Experimental centroided peaks are processed to remove those that have a low intensity or appear in only one MS1 spectrum. Then, MS-Deconv is used to deconvolute MS1 spectra to obtain seed envelopes. (b) Proteoform feature extraction. (1) The reported seed envelopes are ranked based on the sum of the peak intensities of the theoretical envelope. The one with the highest intensity is selected. (2) To extract an envelope set, peaks in the seed theoretical envelope are matched with experimental peaks and extended in both forward and backward directions until no matching experimental peaks are found. (3) The RT boundaries of the reported envelope set are refined if it contains peaks from neighboring envelope sets. (4) The charge state of the envelope set is evaluated and corrected if needed. (5) Once an envelope set is extracted, the neighboring charge states are explored to find other envelope sets in the envelope collection. (6) The experimental peaks included in the envelope collection are removed from the data. The six steps are repeated for the next seed envelope, which has the highest intensity in the remaining seed list. (c) Postprocessing. The precursor masses of reported envelope collections are first refined. Envelope collections are then merged if they have similar precursor masses and similar retention time ranges. Finally, an ECScore is computed for each envelope collection and those with low ECScore are removed.

Many methods have been proposed^11−22^ for peptide feature detection in bottom-up MS (Supporting Table S1), which is similar to proteoform feature detection in top-down MS. These methods are focused on solving three computational problems in feature detection: (1) grouping peak signals with similar *m*/*z* values in consecutive MS1 scans into an *m*/*z* or envelope trace (Figure 1c), (2) splitting a trace into several corresponding to single peptides if the trace contains peak signals from two or more peptides, and (3) evaluating and ranking reported features.

To identify an *m*/*z* or envelope trace, a seed peak or envelope of a feature and its corresponding scan are selected. The peak or envelope is then extended along the RT in both directions until one or several scans do not contain peaks or envelopes with similar *m*/*z* values matched to the seed.^12^

Trace splitting methods can be divided into three groups. The first is to split a trace at the scans lacking matched peaks or envelopes.^12^ In the second approach, the elution profile of a feature is fitted to a distribution or function, such as a Gaussian distribution or wavelet function, and a cutoff signal intensity is used to determine feature boundaries.^15,23^ The third approach is to use a function, such as a Savitzky–Golay filter,^16,19,22^ to smooth a trace locally and use local minima to determine feature boundaries in the trace.

Reported features are in general evaluated by their peak intensities, peak *m*/*z* errors, and RT ranges.^21^ The quality of envelope features is also determined by the similarity of theoretical and experimental isotopic peak intensity distributions.^12^ Recently, deep learning methods have been proposed to evaluate envelope features.^24^

Feature detection in top-down MS is more challenging than in bottom-up MS, as top-down mass spectra tend to have higher charge state ions, more complex isotopic envelopes, and more overlapping envelopes than bottom-up spectra. As a result, feature detection methods designed for bottom-up MS may fail to achieve good performance for top-down MS.

Several methods have been proposed for feature detection in top-down MS, e.g., Xtract,^25,26^ ProMex,^27^ and FlashDeconv.^28^ ProMex uses a greedy algorithm to cluster isotopic envelopes of the same proteoform across MS1 scans. The peak intensities in the experimental envelopes of a proteoform are aggregated to reduce the measurement errors between theoretical and experimental isotopic distributions. The elution profile of each proteoform feature is constructed and smoothed by a Savitzky–Golay filter. Finally, the RT range is obtained using 1% of the apex intensity as the signal intensity cutoff. The quality of each feature is evaluated by a likelihood ratio function based on a Bayesian network model. In FlashDeconv,^28^ candidate features are identified by searching a mass spectrum for peak groups that are generated from proteoform molecules with the same mass and different charge states. The RT range of a feature is determined by a mass trace detection algorithm, in which features are extended along RT, and feature boundaries are found using a smoothing method.^29^ A feature is evaluated by fitting a Gaussian distribution to the peak intensities with different charge states and computing the cosine similarity between the fitted and experimental intensities. The methods in Xtract have not been published.

In this paper, we propose TopFD, a method for proteoform feature detection in top-down MS, in which the functions in MS-Deconv^30^ are employed to identify feature candidates, and RT boundaries of feature signals are determined using local minima of envelope traces. In addition, a neural network model that takes eight attributes of proteoform features as the input was trained for feature evaluation. TopFD was extensively assessed and compared with ProMex,^27^ FlashDeconv,^28^ and Xtract using seven top-down MS data sets (Supporting Methods S1). Experimental results demonstrated that TopFD outperforms these tools in the accuracy, reproducibility of proteoform feature detection, and reproducibility of proteoform quantification.

## Methods

In an LC-MS experiment, the start, apex, and end RTs of a proteoform are determined by the separation column, the experimental parameters, and the chemical and physical properties of the proteoform. An MS1 spectrum collected during the RT range of a proteoform contains peaks of the proteoform, which can be grouped into one or several isotopic envelopes based on their charge states (Figure 1b). The set of all isotopic envelopes of a proteoform with a specific charge state in the LC-MS map is an envelope set (single charge feature) of the proteoform. The collection of the envelope sets of the proteoform for all charge states is the envelope collection (multicharge feature) of the proteoform. The proteoform feature detection problem aims to find all envelope collections in an LC-MS map and report the monoisotopic mass, RT range, and abundance of each envelope collection (Figure 1c).

### Proteoform Feature Detection

Figure 2 shows the overall scheme of TopFD for proteoform feature detection. In preprocessing, TopFD filters out noise peaks in an LC-MS map and then uses the functions in MS-Deconv^30^ to identify experimental isotopic envelopes of proteoforms in single MS1 spectra. A theoretical isotopic envelope is computed for each experimental isotopic envelope using the Averagine model.^31^ The reported isotopic envelopes are ranked based on their total peak intensities and then used iteratively as seed envelopes for feature detection (Supporting Methods S2).

In feature detection, TopFD first extends a seed envelope to neighboring MS1 scans to obtain an envelope set, then the RT boundaries and charge state of the envelope set are adjusted. Next, the envelope set is extended to identify envelope sets with the same precursor mass and neighboring charge states, resulting in an envelope collection. Finally, peaks used in the envelope collection in the LC-MS map will be removed or reduced (Supporting Methods S3).

In postprocessing, the precursor mass of each envelope collection is refined using its isotopic peaks, and then envelope collections with similar precursor masses and RTs are merged. Finally, a neural network model is used to assign an envelope collection score (ECScore) to each envelope collection, and those with low ECScore are removed (Supporting Methods S4).

## Results

### Training the Neural Network Model for ECScore

A set of envelope collections for training ECScore was generated from three top-down MS data sets: one from SW480 cells with triplicates and the other two from breast cancer (BC) samples, each with six replicates (Supporting Methods S1). On average, 21 190 envelope collections were reported from each SW480 replicate and 1765 from each BC replicate (Supporting Table S2) using the methods for envelope collection identification (Figure 2) with the default parameter settings (Supporting Table S3). Note that all envelope collections reported from the three data sets were used for generating training and validation data sets. We labeled the envelope collections identified from the first SW480 replicate, and the first replicate of each BC data set as follows: An envelope collection was labeled negative if it was reported in only the first replicate and labeled positive if it was reported in all of the three SW480 replicates or ≥5 BC replicates. The unlabeled envelope collections were removed. A total of 8579 envelope collections were labeled positive, and 10 876 were negative. We randomly split the envelope collections with a 67:33 ratio into training and validation sets. There was no overlap among the training set, validation set, and test data sets used in the following experiments. We trained the ECScore model using the training set (Methods section), and ECScore achieved a balanced accuracy of 87.03% and the area under the receiver operating characteristic (ROC) curve (AUC) value of 94.18% on the validation data set. The default cutoff of ECScore was set to 0.5 for filtering out low-quality envelope collections because the ECScore distributions of the validation envelope collections show that the cutoff value can efficiently separate positive envelope collections from negative ones (Supporting Figure S1a). With the cutoff of 0.5, the estimated false discovery rate (FDR) of reported envelope collections is 16.4% in the validation set (Supporting Figure S1b), which is acceptable because protein database search-based proteoform identification will remove most false-positive features in downstream analysis. In addition, the FDR may be overestimated due to inaccurate labeling of negative envelope collections.

### Comparison of ECScore and EnvCNN

We compared the accuracy of ECScore and the EnvCNN score^32^ on two top-down MS data sets: one from SW620 cells with three replicates and the other from ovarian cancer (OC) samples with 10 replicates (Supporting Methods S1). Using the methods in the previous section, we labeled the envelope collections reported from the first replicates of the SW620 and OC data. An envelope collection was labeled negative if it was reported in only one replicate and labeled positive if it was reported in all three SW620 replicates or ≥8 OC replicates. This resulted in an SW620 test set of 8376 positive and 3175 negative envelope collections and an OC test set of 6223 positive and 304 negative envelope collections. Because the EnvCNN model takes single isotopic envelopes, not envelope collections, as the input, all test envelope collections were converted to aggregate experiment envelopes (Supporting Methods S3.3), which were used as the input of the EnvCNN model.

ECScore achieved higher ROC AUC values (Supporting Figure S2) than the EnvCNN score on the OC test set (91.52 vs 83.79%) and the SW620 test set (80.56 vs 61.83%). We also compared the rank-sum values of the two scoring functions on the OC and SW620 test sets. To compute the rank-sum of a list of envelope collections, all envelope collections were ranked in the decreasing order of their scores, and the ranks of all positive envelope collections were summed up. ECScore reduced the rank-sum values compared with the EnvCNN score on the OC data set (2.02 × 10^8^ vs 2.18 × 10^8^) and the SW620 data set (1.42 × 10^8^ vs 1.67 × 10^8^).

### Evaluation Using a Protein Mixture

The accuracy of TopFD in determining proteoform monoisotopic masses and charge states was assessed using a top-down LC-MS/MS data of a five-protein mixture: bovine ubiquitin (8559.62 Da), bovine superoxide dismutase (15 581.78 Da), equine myoglobin (16 941.96 Da), bovine trypsinogen (23 965.49 Da), and bovine carbonic anhydrase (29 006.82 Da) (Supporting Methods S1). TopFD identified the proteoform features of all of the five proteins, demonstrating that TopFD can accurately identify proteoform features with a mass <30 kDa (Supporting Tables S4 and S5). The identified features of the last four proteins contained PTMs. The charge states of the seed envelopes of the features ranged from 11 to 33. The errors of reported monoisotopic masses were ≤0.06 Da for the first four proteoforms, and the error of the bovine carbonic anhydrase proteoform was 1.04 Da, which is a common ±1 Da error in spectral deconvolution.

TopFD relies on isotopic peaks of proteoforms for feature detection, so it can deconvolute only isotopically resolvable proteoforms. The *m*/*z* difference between two neighboring isotopic peaks of a proteoform with a charge state *z* is about 1.00235/*z*, where 1.00235 Da is an estimated mass difference between two isotopologues whose numbers of neutrons differ by one.^33^ When the mass spectrometer cannot resolve such two isotopic peaks, TopFD will fail to correctly report the charge state and monoisotopic mass of the proteoform feature.

### Evaluation of Overlapping Features

To evaluate the performance of TopFD on overlapping features, we extracted the peaks of the proteoform feature of bovine ubiquitin in the five-protein mixture data and used the peaks to generate simulated proteoform features with shifts in *m*/*z* value and RT. Specifically, we obtained all peaks in the LC-MS window defined by the *m*/*z* range [693, 697] and RT range [24, 27] min, which contained an envelope set of bovine ubiquitin with charge state 7. The set of all peaks, denoted as *E*_0,0_, was used to generate a total of 90 simulated LC-MS windows *E*_*i*,*j*_ for *i* = 0, ···, 9 and *j* = 1, ··· , 9. To generate *E*_*i*,*j*_ from *E*_0,0_, we shifted the RTs of all peaks by *j* MS1 scans and the *m*/*z* values of all peaks by *i* shift units (each unit is 1.00235/7). That is, the monoisotopic mass of the feature was shifted by 1.00235*i* Da. For each *E*_*i*,*j*_, we generated a simulated LC-MS map containing only peaks in *E*_0,0_ and *E*_*i,j*_, and then merged all peaks with similar *m*/*z* values (with an error tolerance of 0.01 *m*/*z*) in the same scan. The intensity of a merged peak was set to the sum of the intensities of all peaks being merged. As a result, the map contained two overlapping features with known proteoform monoisotopic masses. TopFD successfully identified the monoisotopic masses of the two features (some with ±1 Da or ±2 Da errors) in the LC-MS maps with ≥6 shifted *m*/*z* units or ≥5 shifted scans (Supporting Figure S3), showing that TopFD can identify overlapping features when they are slightly separated by the *m*/*z* value or RT.

### Evaluation of the Artifacts of Reported Proteoform Features

Following the methods in Jeong et al.,^28^ we assessed the quality of proteoform features reported by feature detection tools using three types of artifact masses: low harmonic masses, high harmonic masses, and isotopologues. Incorrect charge state assignments to isotopic envelopes will result in low and high harmonics masses, which are integer fractions and multiples of true masses of proteoforms, respectively. Errors in computing the monoisotopic masses of envelope collections will introduce isotopologues, which are shifted by the mass of one or several neutrons compared with true masses.

An envelope collection *A* is a mass artifact of another envelope collection *B* if (1) the total peak intensity of *B* is higher than *A*, (2) the overlapping RT range of *A* and *B* is larger than 80% of the RT range of *A*, and (3) the monoisotopic mass of *A* is an isotopologue, low harmonic mass, or high harmonic mass of the monoisotopic mass of *B* (Supporting Methods S5). An envelope collection is valid if it is not a mass artifact of another envelope.

**Figure 3:**
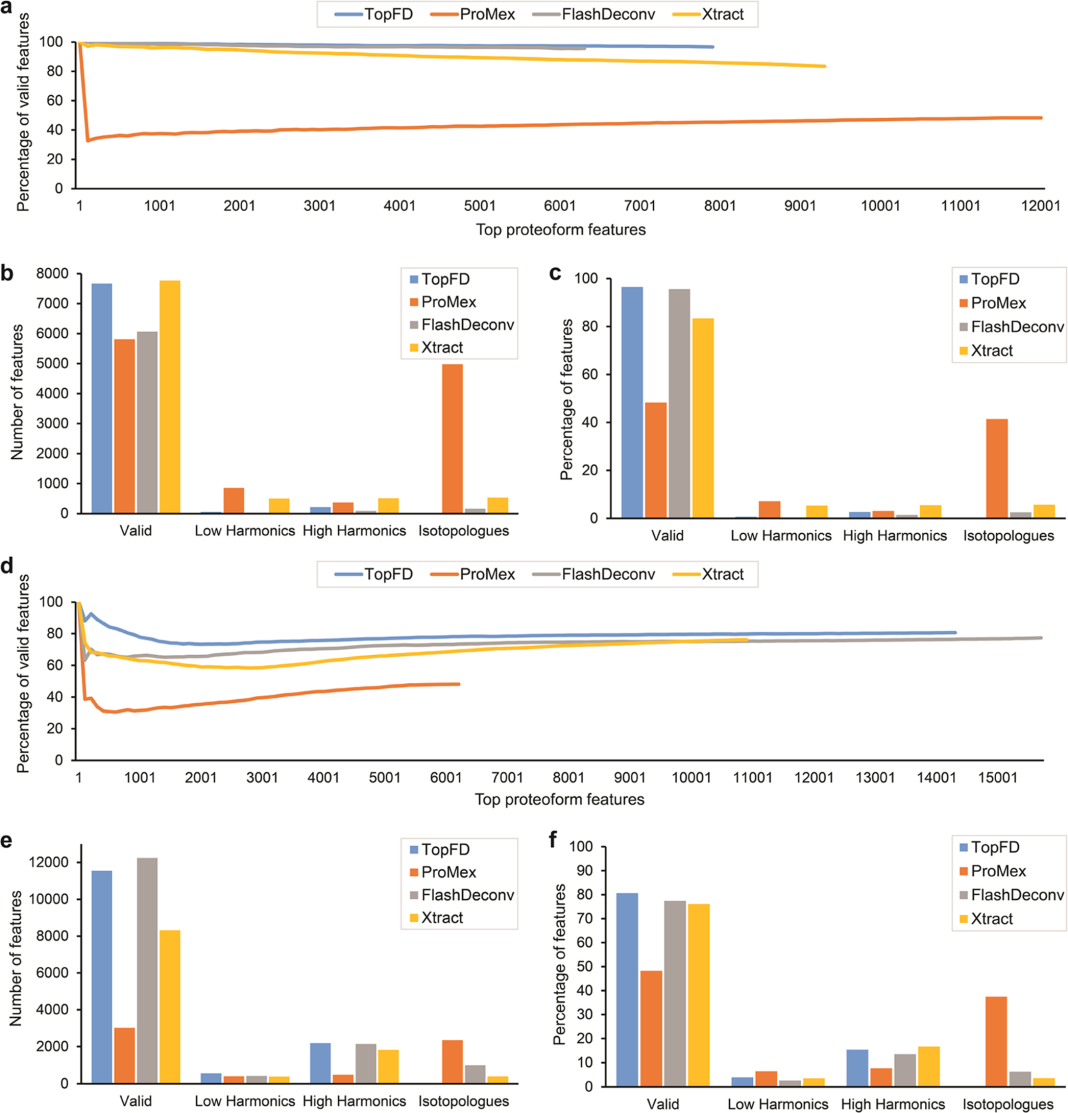
Artifact masses reported by TopFD, ProMex, FlashDeconv, and Xtract from the first replicate of the OC data and the first replicate of the SW620 data. (a) Number of top proteoform features against the percentage of valid features for the first replicate of the OC data. Numbers (b) and percentages (c) of valid, low harmonic, high harmonic masses, and isotopologues in all features reported by the tools from the first replicate of the OC data. (d) Number of top proteoform features against the percentage of valid features for the first replicate of the SW620 data. Numbers (e) and percentages (f) of valid, low harmonic, high harmonic masses, and isotopologues in all features reported by the tools from the first replicate of the SW620 data.

We benchmarked TopFD, ProMex (version 1.1.8082),^27^ FlashDeconv (version 2.0),^28^ and Xtract (Thermo BioPharma Finder 4.1)^25,26^ in the ratio of valid proteoform features using the first OC replicate and the first SW620 replicate. Parameter settings, running times, and numbers of reported proteoform features of the tools are given in Supporting Tables S3, S6–S8, Figure S4, and Table S9, respectively. Valid masses were selected for each software tool separately. After a tool reported a list of proteoform masses from a data set, the method in Jeong et al.^28^ was employed to choose valid masses from the mass list for the tool. For each tool, we ranked the reported proteoform features based on their total peak intensities, obtained the corresponding mass artifacts and valid features, and plotted the ratio of the valid ones against the number of top features. We chose total peak intensities, not software tool-specific scores, to rank features to ensure a fair comparison of the tools. Proteoform features reported by TopFD achieved the best valid ratios (>80%) among the four tools (Figure 3a,d). The valid ratios for Xtract and FlashDeconv are also high, and the distributions of the three types of artifacts are similar for TopFD, FlashDeconv, and Xtract (Figure 3b,c,e,f). ProMex reported many isotopologues, resulting in low valid percentages. We used Fisher’s exact test to compare valid and invalid (mass artifacts) proteoform features reported by TopFD and FlashDeconv as these two outperformed Xtract and ProMex. The *p*-values for the differences between proteoform features reported by TopFD and FlashDeconv are 0.0046 and 4.17 × 10^–12^ for the OC and SW620 data sets, respectively.

### Comparison between Total Ion Currents (TICs) and Feature Intensities

While the TIC of an MS1 scan depicts the number of ions detected by the scan, the total peak intensity of reported proteoform features for an MS1 scan gives the number of ions reported by feature detection tools. These two measurements are expected to be consistent with each other. So following the method in Jeong et al.,^28^ we compared the TICs and total feature intensities reported by the four tools.

To evaluate the correlation between TICs and proteoform feature intensities, we divided the RT range of an MS data set into 1 min RT bins and computed the TIC and total proteoform feature intensity for each bin. The TIC of an RT bin is the sum of the total TIC of all MS1 scans in the bin; the total proteoform feature intensity of an RT bin is the sum of the intensities of the proteoform features whose apex RTs are in the bin. All mass artifacts were removed before the computation of total proteoform feature intensities. The total feature intensities reported by TopFD achieved the best similarity with the TICs on the OC and SW620 data sets (Supporting Figures S5 and S6). For the first OC replicate, the cosine similarity scores between the two measurements are 78.04, 77.46, 77.40, and 61.37% for TopFD, ProMex, FlashDeconv, and Xtract, respectively (Supporting Figure S5). Similarly, for the first SW620 replicate, the cosine similarities are 74.65, 23.61, 73.68, and 67.67% for TopFD, ProMex, FlashDeconv, and Xtract, respectively (Supporting Figure S6).

The software tools reported different feature intensities due to missing envelope sets. We compared the feature intensities reported by the tools in the RT range [114, 115] min of the first OC replicate. TopFD, ProMex, FlashDeconv, and Xtract reported total feature intensities of 1.3 × 10^9^, 1.65 × 10^8^, 7.6 × 10^8^, and 1.49 × 10^8^, respectively. TopFD reported 214 envelope sets (from 52 features) from the RT range, whereas ProMex, FlashDeconv, and Xtract reported 176, 153, and 112 envelope sets, respectively.

### Reproducibility of Proteoform Features in MS Replicates

We benchmarked the feature reproducibility of TopFD against ProMex, FlashDeconv, and Xtract on the OC and SW620 data sets (see the Supporting Material). In MS technical replicates, a true proteoform feature is expected to be observed in all of the replicates, so the frequencies of proteoform features reported from MS technical replicates are a good metric for evaluating proteoform features.^27^ Because mass artifact removal can improve the quality of reported proteoform features, we removed mass artifacts from proteoform features reported by the tools.

We first examined the overlapping features and proteoform mass distributions reported by the four tools in the first OC and SW620 replicates. The four tools reported different numbers of proteoform features from each MS data file (Supporting Figure 7a,b). TopFD, ProMex, FlashDeconv, and Xtract reported 7672, 5811, 6067, and 7773 features in the first OC replicate and 11 552, 3025, 12 240, and 8322 features in the first SW620 replicate, respectively. The two data sets have different distributions of feature masses (Supporting Figure 8a,b). The most observed masses reported by TopFD are between 3 and 4.5 kDa for the OC data and between 1.5 and 3 kDa for the SW620 data. Of the 7672 TopFD features from the OC data, 3233 (42.1%) are shared with all of the other three tools and 6164 (80.1%) are shared with at least one of the three tools. Of the 11 552 TopFD features from the SW620 data, 1535 (13.3%) are shared with all of the other three tools and 7707 (66.7%) are shared with at least one of the three tools. The low overlap for the SW620 data may indicate high false-positive rates of the reported features due to the high complexity of the data.

We further studied the overlapping features and proteoform mass distributions in the same number of top features reported by the four tools in the first OC and SW620 replicates. For each data file, we selected the top *n* features from each of the four feature lists provided by the tools, where *n* is the smallest size among the four feature lists (*n* = 5811 for OC and 3025 for SW620). Note that this method was used to keep only top features for all replicates in the following evaluation of proteoform feature reproducibility. These top features have higher percentages of overlapping features (Supporting Figure 7c,d) compared with all reported features. Of the top TopFD features from the OC data, 2932 (50.5%) are shared with all of the other three tools and 5071 (87.3%) are shared with at least one of the three tools. Of the top TopFD features from the SW620 data, 507 (16.8%) are shared with all of the other three tools and 2382 (78.7%) are shared with at least one of the three tools. The proteoform mass distributions of the top features reported by TopFD (Supporting Figure 8c,d) are similar to those in all of the features.

We investigated the reproducibility of the four tools on the first two replicates of the OC and SW620 data sets. We kept only the top features in each of the four feature lists reported by the tools from each replicate to make sure that the same number of features were used for comparison: OC replicate 1: 5811, OC replicate 2: 5819, SW620 replicate 1: 3025, SW620 replicate 2: 2984. TopFD reported the highest percentage (4781, 83.82%) of proteoform features shared in the two OC replicates compared with ProMex (4327, 74.46%), FlashDeconv (3803, 65.44%), and Xtract (3841, 66.09%). TopFD also outperformed other tools in feature reproductivity on the first two SW620 replicates. A total of 75.86% proteoform features reported by TopFD (2295 out of 3025) were observed in both SW620 replicates, which was better than ProMex (2028, 67.04%), FlashDeconv (1850, 61.15%), and Xtract (1994, 65.19%).

We further extended the feature reproducibility analysis to the 10 replicates of the OC data and three replicates of the SW620 data. Similarly, we kept only the top features in valid feature lists reported by the tools to make sure the same number of features were used for each replicate. On average, 6034 features were used for each of the OC replicates and 3048 for each of the SW620 replicates (Supporting Table S10). For each tool, the features reported from the first replicate (5811 for OC and 3025 for SW620) were compared with those reported from other replicates to obtain their numbers of occurrences. TopFD reported the highest number (3546 out of 5811) of proteoform features reported in all 10 replicates of the OC data set (Supporting Figure S9a) and the highest percentage (77.66%) of proteoform features in 8 or more replicates compared with ProMex (65.96%), FlashDeconv (57.76%), and Xtract (62.14%). Similarly, TopFD outperformed the other tools in feature reproductivity on the SW620 data set (Supporting Figure S9b). A total of 66.47% proteoform features reported by TopFD were observed in all three replicates, which was better than ProMex (57.45%), FlashDeconv (47.80%), and Xtract (55.63%). As ProMex achieved the best performance among the other tools, we utilized Kolmogorov–Smirnov test^34^ to compare the distributions of feature observation frequencies between TopFD and ProMex. The *p*-values are 4.64 × 10^–35^ and 3.87 × 10^–11^ for the distribution differences in the OC and SW620 data sets, respectively, showing that TopFD can report a significantly larger number of reproducible features compared with other tools.

### Quantitative Reproducibility

We benchmarked the four tools in the reproducibility of proteoform abundances using the top valid features (Supporting Table S10) reported from the OC and SW620 data sets. High-accuracy proteoform feature detection is essential for increasing the reproducibility of proteoform abundances measured in MS replicates. To compare the abundance reproducibility between two replicates for a tool, we obtained the overlapping top valid features reported by the tool in the replicates and computed the Pearson correlation coefficient (PCC) of the log-abundances of the overlapping features. We first compared the quantitative reproducibility of the four tools using the first two replicates of the OC and SW620 data sets. For the OC data, TopFD reported a PCC of 98.12% while ProMex, FlashDeconv, and Xtract reported 96.57, 94.97, and 94.37%, respectively. For the SW620 data, TopFD, ProMex, FlashDeconv, and Xtract reported similar PCC values of 94.42, 94.19, 94.70, and 92.13%, respectively. We further extended the PCC analysis to all of the replicates in the OC and SW620 data sets. TopFD reported better proteoform abundance reproducibility compared with other tools, thus indicating good reproducibility in proteoform quantification on the OC (Supporting Figure S10) and SW620 (Supporting Figure S11) data sets.

### Feature Reproducibility in Different Mass Ranges

We first compared the feature reproducibility of the four tools in different mass ranges using the first two replicates of the OC and SW620 data sets. We divided the top valid features reported by each tool into the mass ranges [0, 1.5k], [1.5k, 3k], [3k, 4.5k], [4.5k, 6k], [6k, 7.5k], and [7.5k, 100k] Da. Using the methods described in the previous sections, we compared overlapping proteoform features and proteoform quantitative reproducibility of the four tools in each mass range. TopFD reported the highest overlapping feature ratios in all mass ranges compared with other tools in the two data sets except for the range [7.5k, 100k] in the SW620 (Supporting Figure S12). The reason is that the ratio might not be correctly estimated due to the small number (11) of proteoform features reported by TopFD in the range. TopFD also achieved a slightly better PCC for reported proteoform abundances in most mass ranges in the two data sets (Supporting Figure S13).

### Comparison between Technical Replicates and Biological Replicates

We further compared the feature reproducibility in technical and biological replicates of semen protamine (SP) top-down MS data set (Supporting Methods S1). We used TopFD to identify proteoform features from three runs of the data set: SP11 (technical replicate 1 of biological replicate 1), SP21 (technical replicate 2 of biological replicate 1), and SP12 (technical replicate 1 of biological replicate 2) using the parameters in Supporting Table S3, and mass artifacts were removed from the proteoform features reported by TopFD. The features (1726) reported from SP11 were compared with those reported from SP21 and SP12 separately to evaluate feature reproducibility between technical and biological replicates. TopFD obtained a reproducibility of 51.39% (887 of 1726) for the proteoform features reported from the two technical replicates and a reproducibility of 40.90% (706 of 1726) for features reported from the two biological replicates. The correlation of the proteoform log-abundances reported by TopFD was 90.90% for the technical replicates, which was better than the correlation (80.21%) for the biological replicates.

## Conclusions and Discussion

In this paper, we proposed TopFD, a software tool for top-down MS feature detection that integrates algorithms for proteoform feature detection, feature boundary refinement, and machine learning models for proteoform feature evaluation. Using a standard protein mix, we demonstrated that TopFD can accurately report proteoform features. We further demonstrated TopFD’s ability to parse overlapping envelopes using a simulated data set. An extensive benchmarking of TopFD, ProMex, FlashDeconv, and Xtract using several top-down MS data sets demonstrated that TopFD outperforms other tools in feature accuracy, reproducibility, and feature abundance reproducibility. In comparison with other tools, TopFD also reported fewer artifacts and reported feature intensities had the highest correlation with total ion current.

Accurate proteoform feature detection in top-down MS is essential for proteoform quantification. Proteoform feature detection results show that more than 61% of features reported from one replicate are observed in all three replicates of the SW620 data and all 10 replicates of the OC data, and the PCCs of proteoform abundances between MS replicates are higher than 94%. This level of reproducibility makes it possible to identify differentially expressed proteoforms in two types of samples in proteome-wide top-down MS studies.

Accurate precursor monoisotopic mass calculation in top-down MS is indispensable for identifying PTMs and other alterations in proteoforms. Proteoform feature detection tools often report monoisotopic masses of features with ±1 Da errors due to noise in measured isotopic peak intensities in experimental envelopes and errors in isotopic peak intensities in theoretical envelopes. Following the method in Park et al.,^27^ TopFD aggregates isotopic envelopes in an envelope collection to improve the accuracy of experimental isotopic peak intensities. The errors in theoretical peak intensities are mainly introduced by the difference between the chemical composition of the proteoform and that computed based on the Averagine model.^31^ To address this problem, a postprocessing step can be employed to recalculate the theoretical isotopic peak intensities when the proteoform sequence of the feature is identified and its chemical composition is known.

ECScore in TopFD outperforms the EnvCNN score^32^ for computing confidence scores for identified envelope collections, showing that neural network-based models have the potential to improve the accuracy in proteoform feature detection. ECScore is based on a simple fully connected neural network with eight attributes of envelope collections as the input. As complex neural network models^24^ have been successfully used for peptide feature detections in bottom-up MS, a future research direction is to employ deep learning models to solve various problems in proteoform feature detection, such as feature boundary detection and scores of envelope collections, and use these models to further improve the accuracy in proteoform feature detection.

There are still many challenging problems in proteoform feature detection, like feature boundary detection and the identification of overlapping proteoform features and low abundance features. TopFD identifies overlapping proteoform features by comparing isotopic peak intensities in experimental and theoretical envelopes. If there is a significant difference between the intensities of a pair of matched experimental and theoretical peaks, the experimental peak is treated as an overlapping peak. TopFD relies on seed envelopes for proteoform feature identification and may fail to find seed envelopes for low abundance features. Deep learning models are promising to provide better solutions for identifying overlapping features and low abundance features.

## Data Availability

TopFD is available as part of the TopPIC suite at https://github.com/toppic-suite/toppic-suite/releases/tag/v1.7_beta. The SW480 and SW620 data sets are available at the MassIVE repository (ID: MSV000090488). The data and Python scripts for training the ECScore model and evaluating the performance of the feature extraction tools are available at https://www.toppic.org/software/toppic/topfd_supplemental.html.

## Supporting Information Available

Seven data sets used in the experiments; methods of TopFD; methods for determining artifact masses; ECScore cutoffs and FDRs; comparison of ECScore and EnvCNN; evaluation of TopFD on a protein mixture and overlapping features; comparison of the running times, similarity with TIC, overlapping features, mass distributions, feature and quantitative reproducibility of TopFD, ProMex, FlashDeconv, and Xtract; bottom-up feature detection tools; and parameter settings for TopFD, ProMex, FlashDeconv, and Xtract (PDF)
