# Supplementary material for: TopFD: A Proteoform Feature Detection Tool for Top–Down Proteomics
Source: Anal Chem. 2023 May 17;95(21):8189–96. doi: 10.1021/acs.analchem.2c05244 (PMC10233584; doi:10.1021/acs.analchem.2c05244)
Supplement: Supplementary file 1 — ac2c05244_si_001.pdf [file ac2c05244_si_001.pdf]

Supporting Information

TopFD: A Proteoform Feature Detection Tool for Top-Down Proteomics

Abdul Rehman Basharat[1], Yong Zang[2], Liangliang Sun[3], and Xiaowen Liu[4, *]

[1]Department of BioHealth Informatics, School of Informatics and Computing, Indiana University-Purdue University Indianapolis, Indianapolis, IN, 46202, USA
[2]Department of Biostatistics and Health Data Sciences, Indiana University School of Medicine, Indianapolis, IN, 46202, USA
[3]Department of Chemistry, Michigan State University, East Lansing, MI, 48824, USA
[4]Deming Department of Medicine, Tulane University School of Medicine, New Orleans, LA, 70112, USA

*Correspondence: xwliu@tulane.edu

Table of Content

Supplementary Methods **3**

S1. Data Sets 3

S2. Preprocessing in TopFD 4

S2.1 Data preprocessing *4*

S2.2 Seed envelope identification *4*

S3. Feature detection in TopFD 5

S3.1 Extending a seed envelope to an envelope set *5*

S3.2 Adjusting RT boundaries *6*

S3.3. Correcting charge states *6*

S3.4 Extending an envelope set to an envelope collection *7*

S3.5 Removing envelope collections from experimental data *7*

S4. Postprocessing in TopFD 7

S4.1 Refining monoisotopic masses of envelope collections *7*

S4.2. Merging envelope collections *8*

S4.3. The neural network model for ECScore *8*

S5. Determining artifact masses 8

Supplementary Figures **9**

Supplementary Figure S1. ECScore cutoffs and FDRs. 9

Supplementary Figure S2. Comparison between ECScore and the EnvCNN score on the OC and SW620 test data 9

Supplementary Figure S3. Evaluation of TopFD for the identification of overlapping proteoform features using 90 simulated LC-MS maps 10

Supplementary Figure S4. Running times of TopFD, ProMex, Xtract, and FlashDeconv 10

Supplementary Figure S5. Comparison of TIC and total proteoform feature intensities reported by feature detection tools along the RT for the first OC replicate 11

Supplementary Figure S6. Comparison of TICs and total proteoform feature intensities reported by four feature detection tools along the RT for the first SW620 replicate. 11

Supplementary Figure S7. Venn diagrams showing the overlap in proteoform features reported by TopFD, ProMex, FlashDeconv, and Xtract 12

Supplementary Figure S8. Distributions of proteoform feature masses reported by TopFD, ProMex, FlashDeconv, and Xtract. 13

Supplementary Figure S9. Comparison of the reproducibility of proteoform features reported by TopFD, ProMex, FlashDeconv, and Xtract in MS replicates. 13

Supplementary Figure S10. Quantitative reproducibility of proteoform features reported from the ten replicates of the OC data set. 14

Supplementary Figure S11. Quantitative reproducibility of proteoform features reported from the three replicates of the SW620 data set. 14

Supplementary Figure S12. Comparison of overlapping features reported by TopFD, ProMex, FlashDeconv, and Xtract in different proteoform mass ranges. 15

Supplementary Figure S13. Comparison of the quantitative reproducibility of proteoform features reported by TopFD, ProMex, FlashDeconv, and Xtract in different mass ranges 15

Supplementary Figure S14. An illustration of adjusting the RT boundaries of an envelope set. 15

Supplementary Tables 16

Supplementary Table S1. Summary of bottom-up feature detection tools 16

Supplementary Table S2. Envelope collections reported from the SW480 data set and the two breast cancer data sets 17

Supplementary Table S3. Parameter settings for TopFD 18

Supplementary Table S4. Comparison of theoretical masses and feature masses reported by TopFD for the five proteoforms in the top-down five-protein mixture LC-MS data 19

Supplementary Table S5. Proteoform features reported by TopFD from the top-down five-protein mixture LC-MS data 20

Supplementary Table S6. Parameter settings for ProMex 20

Supplementary Table S7. Parameter settings for FlashDeconv 21

Supplementary Table S8. Parameter settings for Xtract 22

Supplementary Table S9. The numbers of all features, valid features, and mass artifacts reported from the OC and SW620 data sets by TopFD, ProMex, FlashDeconv, and Xtract...23

Supplementary Table S10. The numbers of top valid features kept for comparison of TopFD, ProMex, FlashDeconv, and Xtract in the OC and SW620 replicates......................................24

Supplementary Table S11. Eight input attributions of envelope collections in the neural network model for ECScore 25

Supplementary Methods

S1. Data Sets

Seven top-down MS data sets were used in the experiments. The first two data sets were generated from SW480 and SW620 colorectal cancer (CRC) cells[1] and are available at MassIVE (ID: MSV000090488). Proteoforms extracted from the sample were analyzed using a Thermo Q-Exactive HF mass spectrometer coupled with a 105-minute nanoRPLC separation system. The top 5 precursor ions in each MS1 spectrum were selected from an isolation window of 4 *m*/*z* for MS/MS analysis using higher-energy collisional dissociation (HCD). Both MS1 and MS/MS spectra were acquired at a resolution of 120,000 (at 200 *m*/*z*). Three technical replicates were obtained for each cell line.

The third data set was generated from ovarian cancer (OC) samples[2] and downloaded from MassIVE (ID: MSV000080257). In the experiment, five OC patient samples were pooled, and the extracted proteoforms were analyzed using a Thermo Velos Orbitrap Elite mass spectrometer coupled with a 180-minute LC separation system. The top 4 precursor ions in each MS1 spectrum were selected separately with an isolation window of 10 *m*/*z* for MS analysis using collision-induced dissociation (CID). MS1 and MS/MS spectra were acquired at a resolution of 240,000 and 120,000 (at 400 *m*/*z*), respectively. A total of 10 MS experiment replicates were obtained.

The fourth and fifth data sets were generated from two patient-derived mouse xenografts derived from basal-like and luminal-B human breast cancer samples[3], which were downloaded from the CPTAC data portal (https://cptac-data-portal.georgetown.edu/study-summary/S028). The GELFrEE method[4] was performed for each sample to obtain a fraction containing proteoforms of size up to 30 kDa. Subsequently, six technical replicates were generated for each sample. The samples were analyzed using a Thermo Orbitrap Elite mass spectrometer coupled with an LC system with 90-minute separation. The top two precursor ions in each MS1 spectrum were selected from an isolation window of 15 *m*/*z* for MS/MS analysis using HCD. MS1 and MS/MS spectra were acquired at a resolution of 120,000 and 60,000 (at 400 *m*/*z*), respectively.

The sixth data set was generated from two human semen samples[5] (PRIDE repository ID: PXD024405). Protamine proteoforms were extracted from the samples and analyzed using a 60-minute HPLC separation system coupled with a Thermo Orbitrap Fusion Lumos mass spectrometer. The most intense precursor ions in each MS1 spectrum were selected from an isolation window of 0.7 *m*/*z* for MS/MS analysis using electron transfer dissociation (ETD) with a cycle time of 3 seconds. Both MS1 and MS/MS spectra were acquired at a resolution of 120,000 (at 200 *m*/*z*). Two technical replicates were acquired for each of the two samples.

The seventh data set was generated using a mixture of five proteins containing bovine carbonic anhydrase (Sigma C2624), equine myoglobin (Sigma M5696), bovine trypsinogen (Sigma T1143), bovine ubiquitin (Sigma U6253), and bovine superoxide dismutase, in which bovine superoxide dismutase was present as a contaminant in bovine carbonic anhydrase[6]. The samples were analyzed using a 50-minute LC separation system coupled with a Thermo Velos Orbitrap Elite mass spectrometer. The top precursor ion in each MS1 spectrum was selected from an isolation window of 15 *m*/*z* for MS/MS analysis using HCD. The MS1 and MS/MS spectra were collected at a resolution of 120,000 and 60,000 (at 400 *m*/*z*), respectively.

S2. Preprocessing in TopFD

S2.1 Data preprocessing

MsConvert[7] was used to convert raw files into centroided mzML files. Two methods were used to filter out noise peaks in MS1 spectra to speed up proteoform feature detection. The first filtering method was based on peak intensities, in which peaks with intensity lower than a cutoff intensity were removed because most of them do not provide valuable information for feature detection. To obtain the cutoff intensity, a histogram of the intensities of all MS1 peaks in the data file was generated, and the noise intensity level, denoted by h, was set to the middle value of the bin with the highest frequency[8], and the cutoff intensity was set to $3h$. The second filtering method was based on the number of consecutive spectra in which a peak is observed. Peaks that appear in only one MS1 spectrum, not several consecutive MS1 spectra, tend to be noise ones. Therefore, a peak in an MS1 spectrum was removed if it was not observed in its neighboring MS1 scans within an *m*/*z* error tolerance of 0.01.

S2.2 Seed envelope identification

We obtained isotopic envelopes of proteoforms from single spectra and then used them as seeds to find envelope sets and envelope collections. Experimental isotopic envelopes in single MS1 spectra were identified based on the methods in MS-Deconv[8, 9] with eight steps. (1) A peak in the spectrum is selected as the base peak of the envelope. (2) A theoretical isotopic distribution is computed using the Averagine model[10] with a given charge state so that the *m*/*z* value of the highest intensity peak in the envelope equals the *m*/*z* value of the base peak. (3) Peaks in the theoretical distribution are matched to those in the spectrum by comparing their *m*/*z* values with an error tolerance (0.02 in the experiments). The set of matched experimental peaks is reported as an experimental isotopic envelope. (4) A theoretical envelope is obtained by scaling the peak intensities of theoretical distribution so that the sum of the intensities of the top three peaks in the theoretical envelope is the same as that of the top three experimental peaks. (5) The theoretical and experimental envelope pair is scored using the default scoring function in MS-Deconv, and

its monoisotopic mass is computed. (6) Peaks in the envelope pair are removed if their scaled theoretical intensities are lower than a cutoff intensity, which is set to the intensity of *3h*. (7) After all candidate envelopes are generated from the spectrum, a dynamic programming method is used to report a group of theoretical and experimental envelope pairs that fit the spectrum. (8) The envelope pairs are further filtered using a cutoff value (0.5 in the experiments) for the Pearson correlation coefficient (PCC) between the peak intensities of theoretical and experimental envelopes.

S3. Feature detection in TopFD

We ranked the experimental and theoretical envelope pairs reported from all MS1 spectra in an LC-MS run in the decreasing order of the total peak intensity, which is the sum of the peak intensities of the theoretical envelope. The theoretical envelope with the highest intensity was selected as the first seed envelope, which was then extended to neighboring scans to obtain an envelope set. Theoretical envelopes were used for the extension because they tend to have fewer errors in *m*/*z* values and peak intensities than experimental envelopes.

S3.1 Extending a seed envelope to an envelope set

To obtain an envelope set, a seed envelope E of a proteoform was matched to experimental peaks in its neighboring spectra to extend the RT range of the proteoform. Let $S_1, \ldots, S_{i-1}, S_i, S_{i+1}, \ldots, S_n$ be all MS1 spectra in the increasing order of RT, in which the seed spectrum S_i contained the seed envelope E. We first checked if the spectrum S_{i-1} contained a matched experimental envelope of E. The isotopic peaks in E were matched to the experimental peaks in the spectrum to obtain an experimental envelope with an *m*/*z* error tolerance of 0.008. If two or more experimental peaks were matched to one theoretical peak, the one with the highest intensity was selected. Peaks in E were scaled to fit the peak intensities of the experimental peaks using the method in Section S2.2. The scaled peaks in E with an intensity lower than the cutoff intensity of $3h$ (see Section S2.1) were removed from the envelope along with the corresponding matched experimental peaks.

An experimental envelope was matched to the theoretical one if at least two of the three highest theoretical peaks matched experimental peaks. We searched for matched experimental envelopes in the neighboring spectra $S_{i-1}, \ldots, S_1$ until we found two continuous spectra without a matched experimental envelope. The extension was also performed for the other direction in the neighboring spectra $S_{i+1}, \ldots, S_n$.

The RTs of the first and last spectra reported by the extension method are called the initial start and end RTs of the proteoform, respectively. If a spectrum in the initial RT range contains a matched envelope, the corresponding trace intensity value is the sum of the intensities of the top

three highest scaled theoretical peaks and 0 otherwise. The trace intensities of all MS1 spectra in the initial RT range are called the extracted envelope chromatogram (XEC) of the seed envelope.

S3.2 Adjusting RT boundaries

XECs of envelope sets were smoothed using a moving average filter with a window size of 2. Let t_c be the RT of a seed scan and t_s be the start RT of an envelope set. To adjust the start RT, we found all local minima in the XEC between t_s and t_c and ranked them in increasing order of intensity. Let t_{min} be the RT with the lowest XEC value i_{min}. If there was a local maximum with RT $t_{\max}$ and trace intensity $i_{\max}$ such that $i_{\max} > 2.5i_{\min}$ and $t_{\min}$ was between $t_{\max}$ and t_c ($t_s < t_{\max} < t_{\min} < t_c$), then the start RT t_s was set to t_{min} (Supplementary Fig. S14). The process was repeated until all the local minima had been checked. The process was performed to adjust the start and end RTs of reported envelope sets. This allowed us to fix errors in RT boundaries when the extended envelope set contained peaks from two or more neighboring envelope sets. All matched experimental envelopes in the adjusted RT range were reported as an envelope set of the proteoform.

S3.3. Correcting charge states

Because of noise peaks, some seed envelopes reported from single spectra had an incorrect charge state. To correct charge states, we summed up peak signals from several scans in an envelope set to obtain a better signal-to-noise ratio of peaks. For each peak in a seed envelope, the corresponding aggregate envelope peak was obtained by summing up the intensities of matched experimental peaks across all spectra within the RT range of the envelope set.

We used aggregated envelopes to fix one common type of error in charge states, in which a charge state c is mistakenly reported as charge state $2c$. This type of error is called a double charge error. The main reason for double charge errors is that some noise peaks are randomly matched to theoretical peaks with charge state $2c$.

The peaks in the aggregated envelope were ranked in the increasing order of their m/z values. The sums of even and odd index peaks were obtained for both theoretical and aggregate experimental envelopes. In an envelope with a double charge error, the peaks with odd or even indices are usually caused by noise peaks, which are characterized by their low intensities. Let A_e and A_o be the sum of the intensities of even and odd aggregate experimental peaks, respectively. Let B_e and B_o be the sum of intensities of even and odd aggregate theoretical peaks, respectively. The two ratios A_e/B_e and A_o/B_o tend to be significantly different for envelopes with double charge errors. So, we calculated the log ratio with base 10 of the two ratios for each reported envelope set. If the absolute value of the log ratio was greater than 0.4, the charge state

of the seed envelope was halved, and the new seed envelope was used to obtain an envelope set.

S3.4 Extending an envelope set to an envelope collection

After an envelope set with charge state c was reported, an envelope collection was obtained by exploring the neighboring charge states to find isotopic envelopes with the same monoisotopic mass. To find an envelope set with charge state c-1, the theoretical envelope E_{c-1} for charge state c-1 was obtained using the seed theoretical envelope of the envelope set with charge state c. Next, we extended E_{c-1} to obtain the start and end RTs using the methods in the previous section. If we failed to find at least two matched experimental peaks for the top three highest theoretical peaks in E_{c-1} in spectra S_{i-1}, S_i, and S_{i+1}, then the envelope set for charge state c-1 was set to empty. We searched for envelope sets with charge states c-1, c-2, …,1 until two continuous empty envelope sets were found. Similarly, envelope sets were searched for charge states c+1, c+2 … until two continuous empty envelope sets were found. All identified non-empty envelope sets were added to the envelope collection.

S3.5 Removing envelope collections from experimental data

To identify overlapping peaks shared by multiple envelope collections, we scaled peaks in a seed envelope to fit the peak intensities of its matched experimental envelope (see Section S2.2). If the intensity of an experimental peak was at least 4 times higher than that of the corresponding scaled theoretical peak, the peak was considered an overlapping one; otherwise, non-overlapping. To remove an envelope collection, the intensity of an overlapping experimental peak was reduced by the intensity of its matched theoretical peak, and non-overlapping experimental peaks were removed directly.

S4. Postprocessing in TopFD

S4.1 Refining monoisotopic masses of envelope collections

For an experimental peak p in an envelope collection, the m/z error between p and its matched theoretical peak is represented by $e(p)$ and the intensity of its matched theoretical peak is represented by $h(p)$. The weighted average m/z error of all peaks p in the envelope collection is $\frac{\sum_p e(p)h(p)}{\sum_p h(p)}$, and the weighted average mass error of the envelope collection is the product of the average m/z error and the charge state of the seed envelope. The refined monoisotopic mass of an envelope collection was obtained by subtracting the weighted average error mass from its original monoisotopic mass.

S4.2. Merging envelope collections

Once envelope collection F was reported, we checked if it could be merged with another envelope collection. Two envelope collections were merged if (1) the difference between their masses was within $[-1.00235-\epsilon,-1.00235+\epsilon]$, $[-\epsilon,\epsilon]$, $[1.00235-\epsilon,1.00235+\epsilon]$, where ϵ was an error tolerance of 10 ppm and 1.00235 Da is an estimate of the mass difference of neighboring isotopic peaks in an envelope[11], (2) their RT ranges overlap was more than 80% of F, and (3) their change states ranges were not separated by more than 2 charge states.

S4.3. The neural network model for ECScore

The neural network model for ECScore takes eight attributes of an envelope collection as the input (Supplementary Table S11). The neural network model consists of four hidden layers (200 neurons in each layer) and an output layer. The activation function is the Leaky Rectified Linear Unit with a negative slope coefficient of 0.05 for the hidden layers and the sigmoid function for the output layer. L1 kernel regularization is applied to hidden layers with a regularization factor of 1×10^{-6}. The neural network model was implemented using TensorFlow (version 2.7.0). In model training, the loss function was binary cross-entropy and the Adam optimizer[12] with a learning rate of 1×10^{-5} was used. The training process was stopped if the validation loss did not improve for 30 epochs, and the model with the smallest validation loss was reported. To deal with the class imbalance problem in training data, class weighting by the inverse class frequency was used.

S5. Determining artifact masses

For a mass x and a maximum shifted mass of 10 neutrons, the set X of shifted and unshifted masses of x consists of 21 masses x+1.00235d for d=-10, -9, …,10, where 1.00235 Da is an estimated mass difference between two isotopologues introduced by a neutron[11]. A mass y is an isotopologue of mass x if y matches a mass in X with an error tolerance of 10 ppm. And y is a low (high) harmonic mass of x if the mass yc (y/c) matches a mass in X with an error tolerance of 10 ppm, where c is an integer.

Supplementary Figures

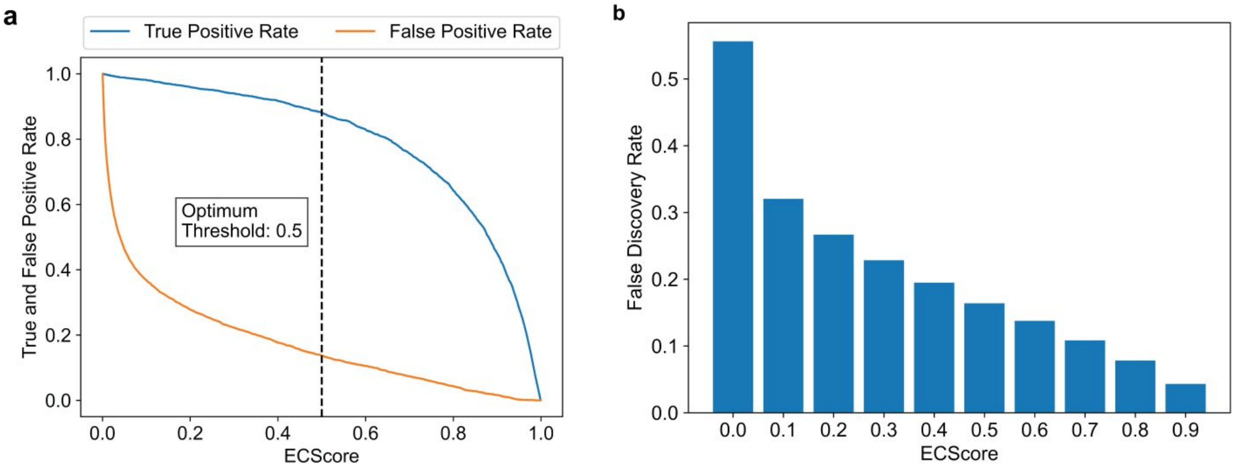

Supplementary Figure S1. ECScore cutoffs and FDRs. (a) True positive and false positive rates of envelope collections in the validation data set for each ECScore cutoff. The maximum difference between the true positive and false positive rates is obtained with a cutoff of 0.488. The value of 0.5 (rounded value of 0.488) is chosen as the default cutoff of ECScore. (b) False discovery rate (FDR) of envelope collections in the validation data set for each ECScore cutoff. The estimated FDR for the cutoff 0.5 is 16.4%.

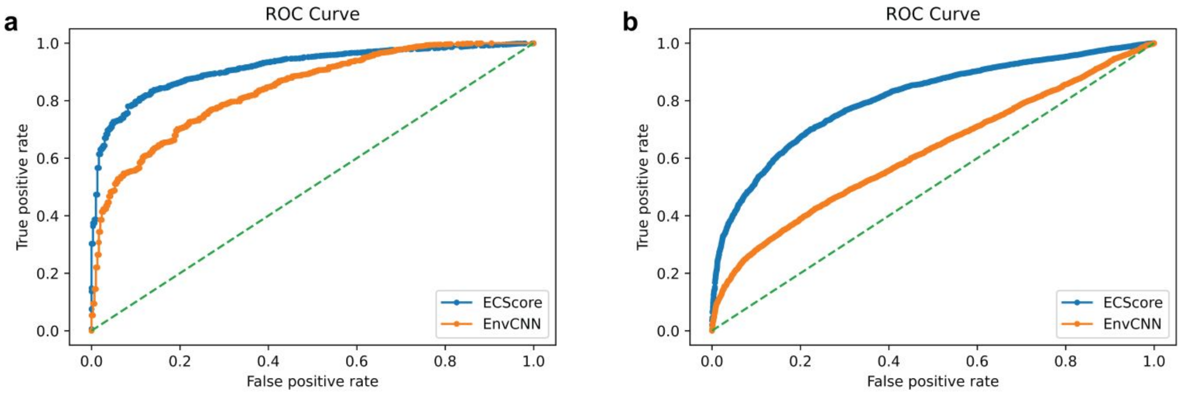

Supplementary Figure S2. Comparison between ECScore and the EnvCNN score on the OC and SW620 test data**.** (a) ROC curves on the OC test envelope collections. (b) ROC curves on the SW620 test envelope collections.

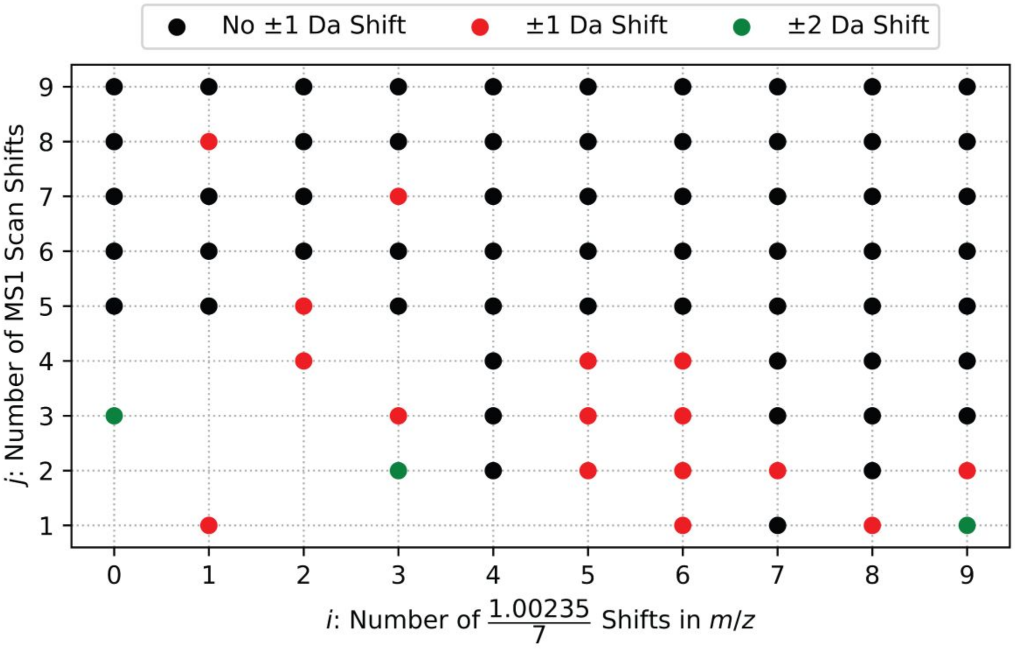

Supplementary Figure S3. Evaluation of TopFD for the identification of overlapping proteoform features using 90 simulated LC-MS maps $M_{i,j}$ (i = 0,1,…,9 and j = 1,2,..,9). Each simulated LC-MS map $M_{i,j}$ contains a proteoform feature (charge 7) of bovine ubiquitin and a shifted version of the feature, in which the *m*/*z* values of the peaks are shifted by *i* shift units (each unit is 1.00235/7) and the retention times of the peaks are shifted by *j* MS1 scans. Each dot represents an LC-MS map for which the two proteoform features are identified by TopFD. The color of each dot indicates the maximum error in the reported two monoisotopic masses of the features: no ± 1 Da shift (black), ± 1 Da shift (red), and ± 2 Da shift (green).

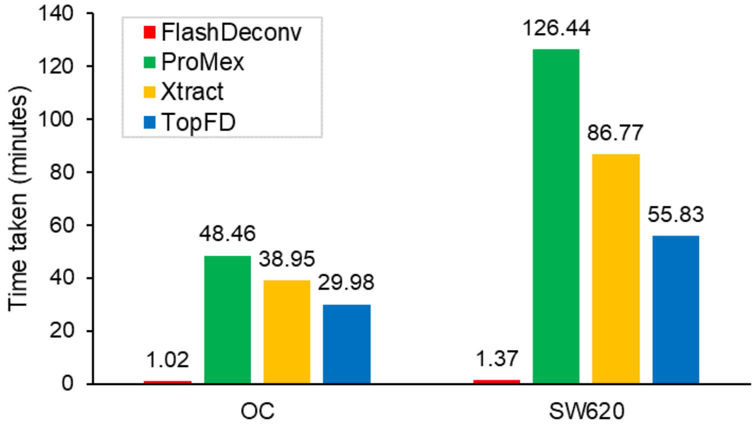

Supplementary Figure S4. Running times of TopFD, ProMex, Xtract, and FlashDeconv on the first OC replicate and the first SW620 replicate. The running time of each tool was obtained on a desktop computer with an Intel® Core™ i7-8700 @ 3.2GHz CPU and 16 GB RAM using 1 CPU thread. Only MS1 spectra were deconvoluted and MS/MS spectra were not deconvoluted in the test.

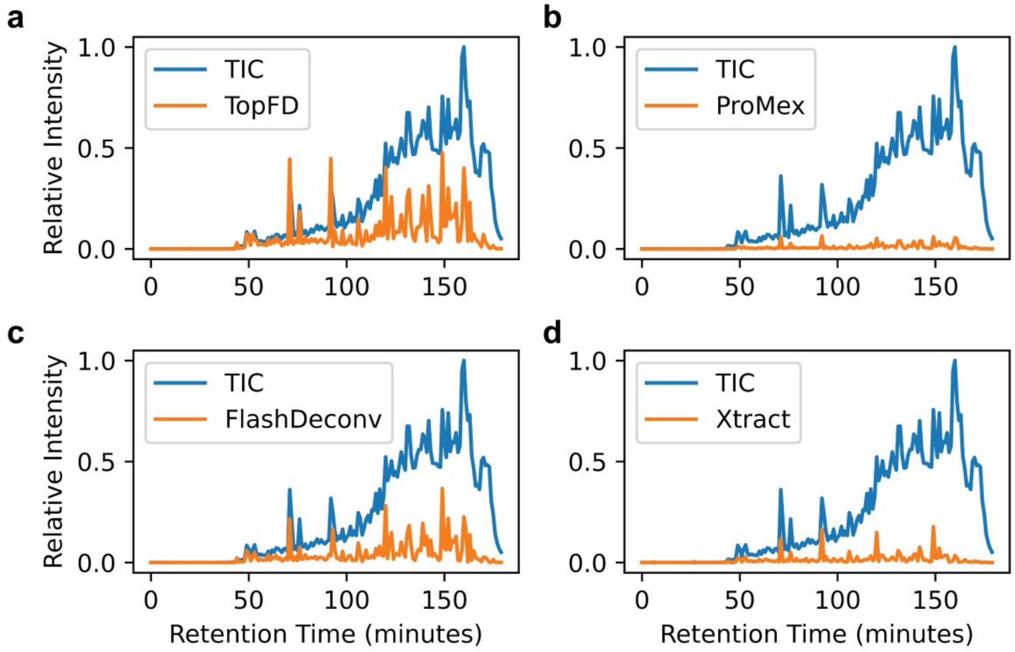

Supplementary Figure S5. Comparison of TIC and total proteoform feature intensities reported by feature detection tools along the RT for the first OC replicate**.** (a) TopFD, (b) ProMex, (c) FlashDeconv, and (d) Xtract. The RT range of the MS data is divided into 1-minute RT bins. The TIC and total proteoform feature intensity in each bin are normalized by dividing them by the maximum TIC value.

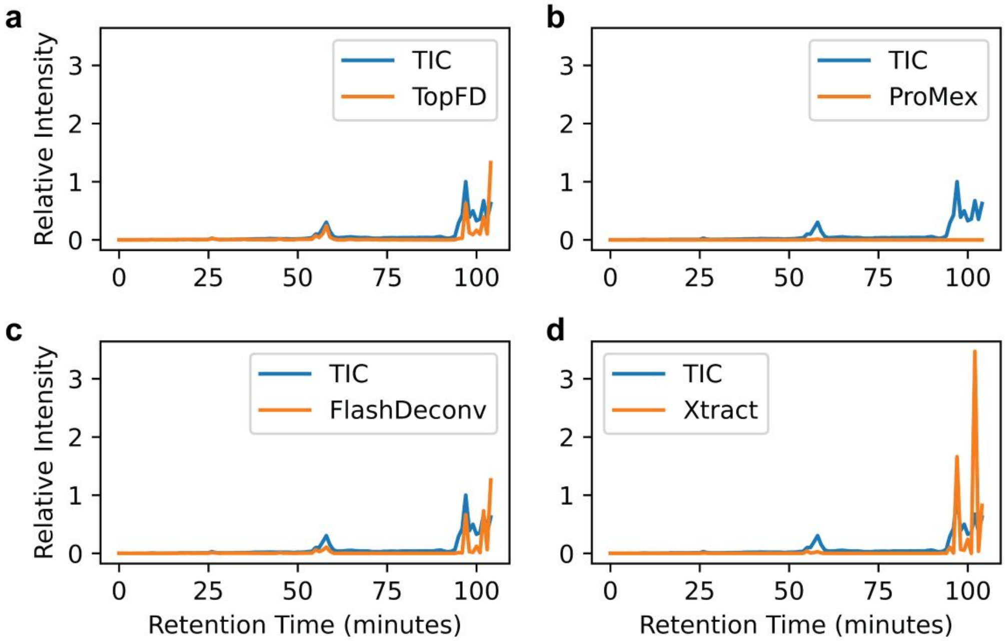

Supplementary Figure S6. Comparison of TICs and total proteoform feature intensities reported by four feature detection tools along the RT for the first SW620 replicate. (a) TopFD, (b) ProMex, (c) FlashDeconv, and (d) Xtract. The RT range of the MS data is divided into 1-minute RT bins. The TICs and total proteoform feature intensities are normalized by dividing them by the maximum TIC value.

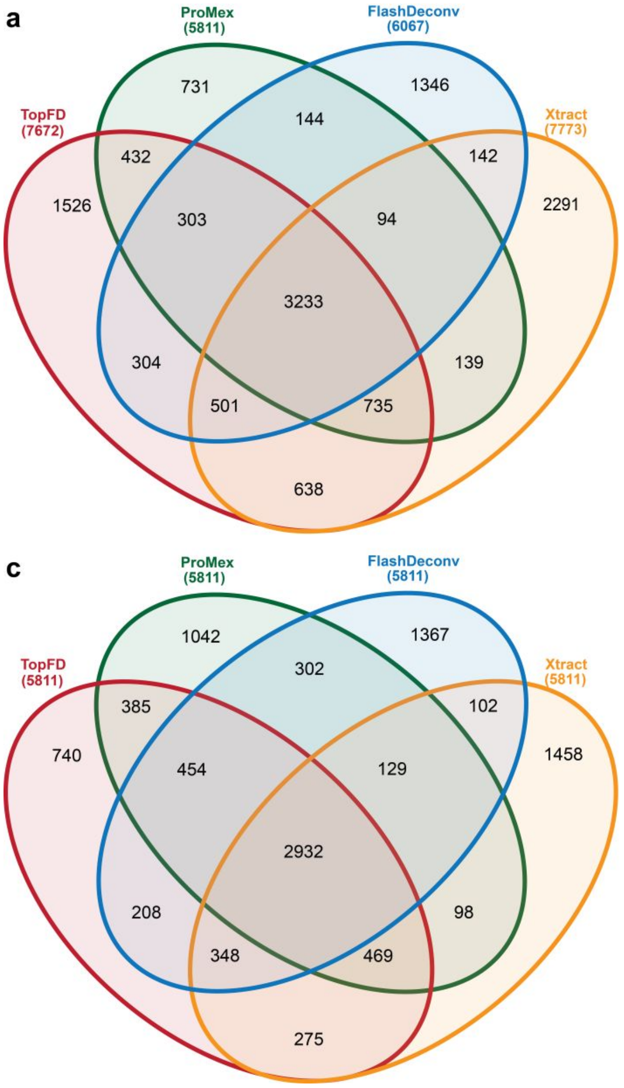

Supplementary Figure S7. Venn diagrams showing the overlap in proteoform features reported by TopFD, ProMex, FlashDeconv, and Xtract. (a) All features reported in the first OC replicate. (b) All features reported in the first SW620 replicate. (c) The top 5811 features reported in the first OC replicate. (d) The top 3025 features reported in the first SW620 replicate.

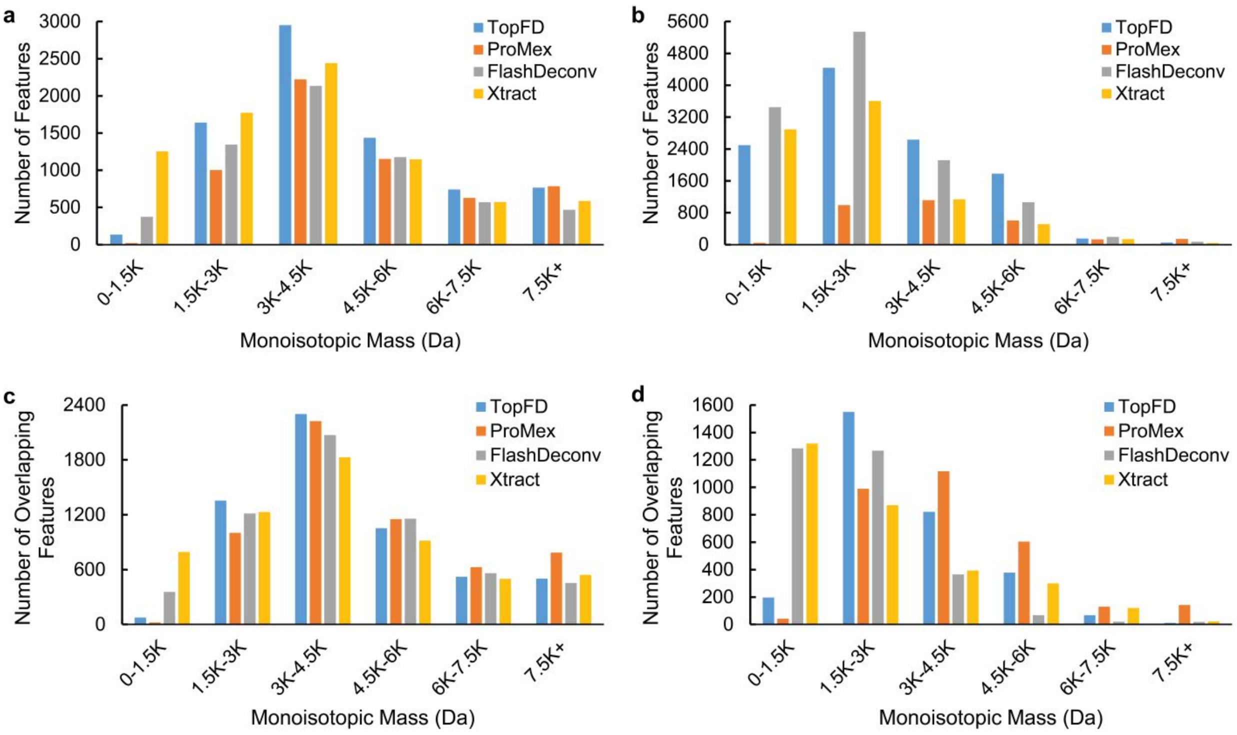

Supplementary Figure S8. Distributions of proteoform feature masses reported by TopFD, ProMex, FlashDeconv, and Xtract. (a) All features reported in the first OC replicate: TopFD: 7672, ProMex: 5811, FlashDeconv: 6067, and Xtract: 7773. (b) All features reported in the first SW620 replicate: TopFD: 11552, ProMex: 3025, FlashDeconv: 12240, and Xtract: 8322. (c) The top 5811 features reported in the first OC replicate. (d) The top 3025 features reported in the first SW620 replicate.

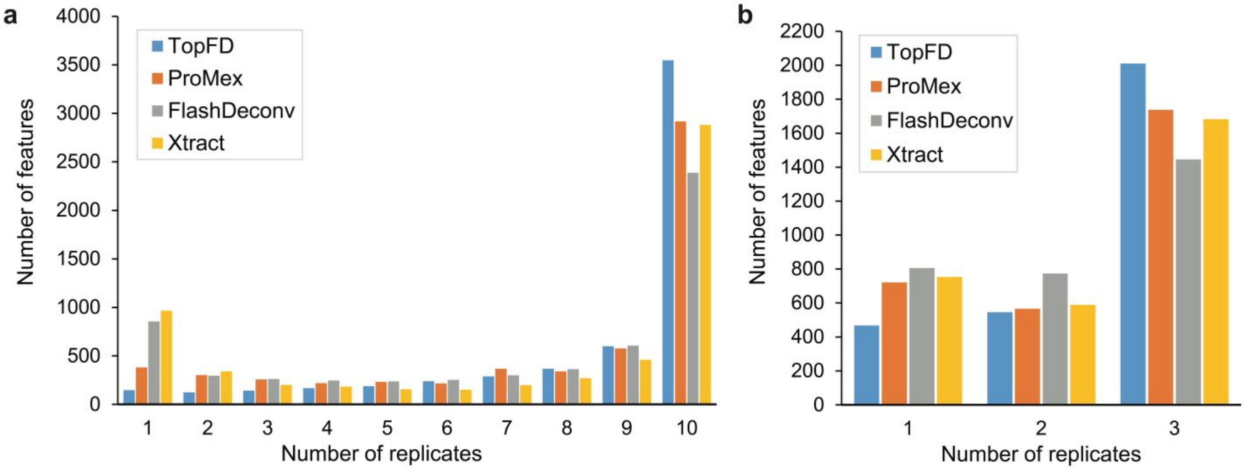

Supplementary Figure S9. Comparison of the reproducibility of proteoform features reported by TopFD, ProMex, FlashDeconv, and Xtract in MS replicates. (a) The frequencies of feature observations in the OC data set for the 5,811 features reported from the first replicate. (b) The frequencies of feature observations in the SW620 data set for the 3,025 features reported from the first replicate.

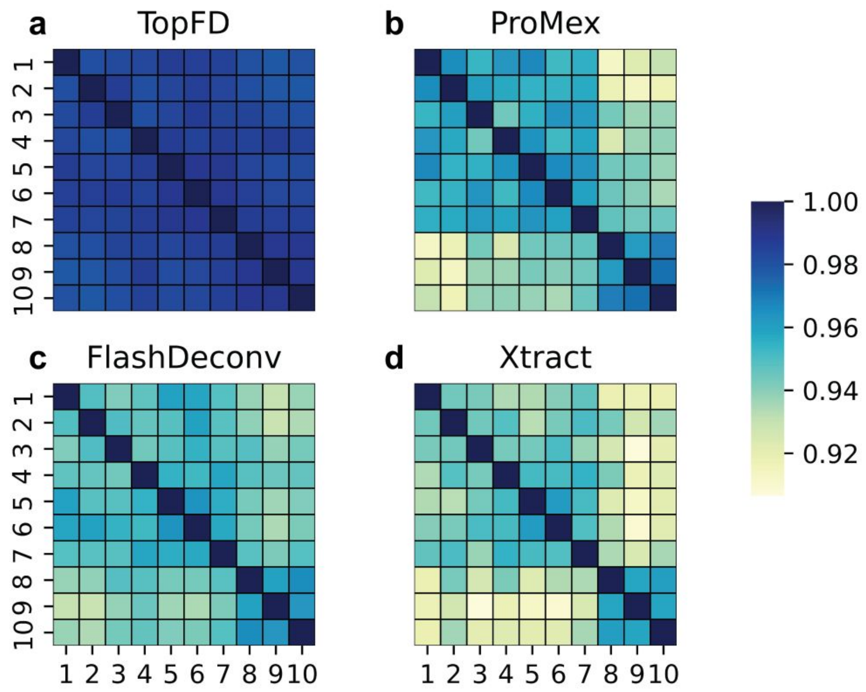

Supplementary Figure S10. Quantitative reproducibility of proteoform features reported from the ten replicates of the OC data set. The PCC between the log-abundances of proteoform features is obtained for each replicate pair for (a) TopFD, (b) ProMex, (c) FlashDeconv, and (d) Xtract.

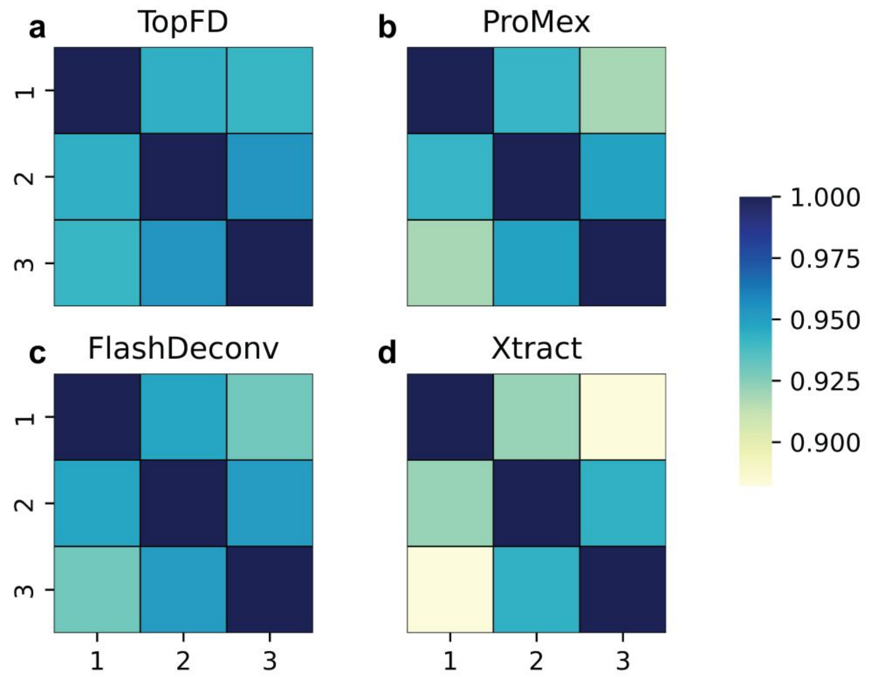

Supplementary Figure S11. Quantitative reproducibility of proteoform features reported from the three replicates of the SW620 data set. The PCC between the log-abundances of proteoform features is obtained for each replicate pair for (a) TopFD, (b) ProMex, (c) FlashDeconv, and (d) Xtract.

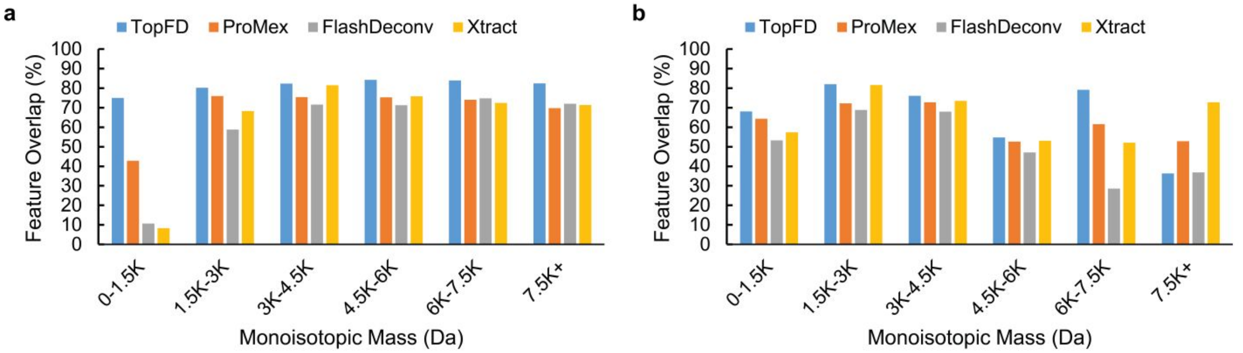

Supplementary Figure S12. Comparison of overlapping features reported by TopFD, ProMex, FlashDeconv, and Xtract in different proteoform mass ranges. Percentages of overlapping features in the first two replicates with respect to the features in the first replicate are computed for each mass range for the (a) OC, and (b) SW620 data sets.

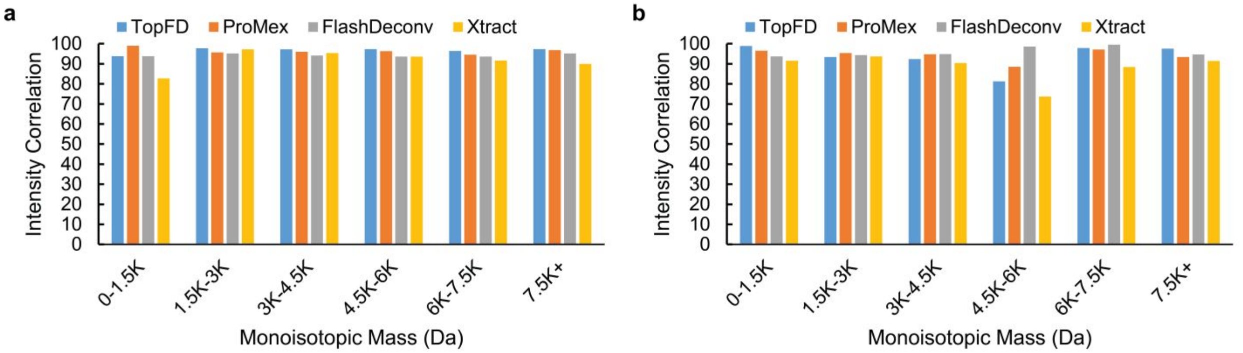

Supplementary Figure S13. Comparison of the quantitative reproducibility of proteoform features reported by TopFD, ProMex, FlashDeconv, and Xtract in different mass ranges on the first two replicates of the (a) OC and (b) SW620 data sets. The PCC is computed using the log-abundances of proteoform features shared by the first two replicates in each mass range.

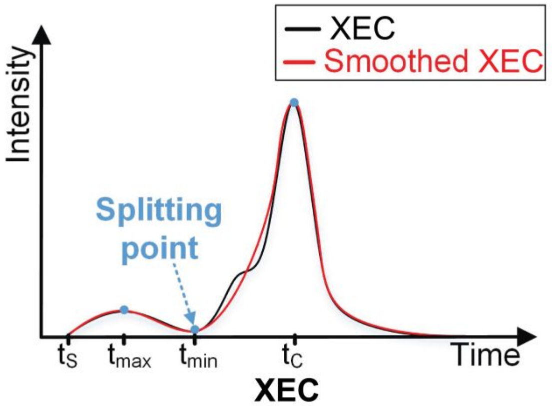

Supplementary Figure S14. An illustration of adjusting the RT boundaries of an envelope set. The XEC of the envelope set contains peaks from two neighboring envelope sets. The XEC is smoothed by employing a moving average filter with a window of 2. t_C is the RT of the spectrum with the seed envelope, and t_S is the original start RT of the envelope set. A local minimum is located at t_{min} and a local maximum is located at t_{max}. The intensity ratio of XEC at t_{max} and t_{min} is greater than 2.5, so the start RT is adjusted to t_{min}.

Supplementary Tables

Supplementary Table S1. Summary of bottom-up feature detection tools

Tool	Description
msInspect[13]	msInspect identifies candidate peaks for feature detection by locating local maxima in each scan. Peaks that elute over several spectra in an LC-MS map are extracted. Using the peptide isotopic distribution, the co-eluting peaks are grouped to report a peptide feature. Kullback–Leibler divergence is used to evaluate the similarity between observed and experimental isotopic distributions.
centWave[14]	centWave uses each peak in experimental data as a candidate seed peak. If a peak in a spectrum is observed in neighboring spectra within a certain *m*/*z* range, these peaks are grouped to obtain a mass trace. A continuous wavelet transform is applied to each mass trace to determine its retention time boundaries.
MaxQuant[15]	In MaxQuant, candidate seed peaks are obtained by finding local intensity maxima in each spectrum. A Gaussian distribution is fitted to the seed peak and other peaks with similar *m*/*z* values in the scan to obtain an *m*/*z*-intensity curve. Afterward, these *m*/*z*-intensity curves are connected in neighboring scans based on their central positions to get the 3-dimensional retention time profile of the feature. Finally, deconvolution is performed based on 3-dimensional retention time profiles extracted from the LC-MS map.
Dinosaur[16]	Dinosaur collects centroided peaks with similar *m*/*z* values in consecutive spectra to build mass traces. Subsequently, deconvolution is performed to obtain peptide features.
DeepIso[17]	DeepIso is comprised of two deep-learning-based modules. The first module scans the experimental LC-MS map along the retention time axis and determines the charge state and retention time range of a feature. The second module scans the experimental LC-MS map along the *m*/*z* axis to group peaks that belong to the same isotopic distribution to report peptide features.
MSTracer[18]	MSTracer extracts mass traces and then groups mass traces whose local maxima are located at similar RT and whose intensities match the isotopic distribution of a peptide. A support vector regression model is employed to evaluate overlapping mass trace groups and report the best-scoring one. A deep-learning model is used to report the quality score for each peptide feature.

Supplementary Table S2. Envelope collections reported from the SW480 data set and the two breast cancer data sets

Data	Number of Seed Envelopes	Number of Envelope Collections
SW480 colorectal cell line data – Replicate 1	471,330	20,218
SW480 colorectal cell line data – Replicate 2	524,968	22,623
SW480 colorectal cell line data – Replicate 3	520,505	20,731
Basal-like breast cancer data – Replicate 1	67,730	1,785
Basal-like breast cancer data – Replicate 2	65,058	1,664
Basal-like breast cancer data – Replicate 3	68,751	1,820
Basal-like breast cancer data – Replicate 4	69,213	1,863
Basal-like breast cancer data – Replicate 5	63,547	1,684
Basal-like breast cancer data – Replicate 6	65,159	1,823
Luminal-B breast cancer data – Replicate 1	64,141	1,727
Luminal-B breast cancer data – Replicate 2	64,466	1,902
Luminal-B breast cancer data – Replicate 3	71,712	1,767
Luminal-B breast cancer data – Replicate 4	67,956	1,833
Luminal-B breast cancer data – Replicate 5	69,770	1,554
Luminal-B breast cancer data – Replicate 6	68,126	1,758

Supplementary Table S3. Parameter settings for TopFD

**Input Parameter**	**Value**
Maximum charge	60
Maximum mass	100,000 Da
MS1 signal noise ratio in MS-Deconv	3.0
M/*z* error tolerance in MS-Deconv	0.02
Disable final filtering in MS-Deconv	True
Use EnvCNN score in MS-Deconv	False
PCC cutoff for seed envelopes	0.5
Signal noise ratio for peak filtering	3.0
m/*z* error tolerance for peak filtering	0.01
m/z error tolerance for envelope extension	0.008
ECScore cutoff	0.5*

ECScore cutoff was not used in the generation of training data sets for the neural network model

Supplementary Table S4. Comparison of theoretical masses and feature masses reported by TopFD for the five proteoforms in the top-down five-protein mixture LC-MS data

Protein Name	Reported Mass (Da)	Expected Mass (Da)	Mass Error (Da)	Modifications
	**Sequence**			
Bovine Ubiquitin	8,559.64	8,559.62	0.02	No modification
	MQIFVKTLTGKTITLEVEPSDTIENVKAKIQDKEGIPPDQQRLIFAGKQLEDGRTLSDYNIQKESTLHLVLRLRGG			
Bovine Superoxide Dismutase	15,581.83	15,581.78	0.05	N-terminal acetylation after the initiator methionine is removed and one cysteine bridge*
	[*Acetyl*]ATKAVCVLKGDGPVQGTIHFEAKGDTVVVTGSITGLTEGDHGFHVHQFGDNTQG(C)[*HydrogenLoss*]TSAGPHFNPLSKKHGGPKDEERHVGDLGNVTADKNGVAIVDIVDPLISLSGEYSIIGRTMVVHEKPDDLGRGGNEESTKTGNAGSRLA(C)[*HydrogenLoss*]GVIGIAK			
Equine Myoglobin	16,941.02	16,940.96	0.06	Initiator methionine is removed
	GLSDGEWQQVLNVWGKVEADIAGHGQEVLIRLFTGHPETLEKFDKFKHLKTEAEMKASEDLKKHGTVVLTALGGILKKKGHHEAELKPLAQSHATKHKIPIKYLEFISDAIIHVLHSKHPGDFGADAQGAMTKALELFRNDIAAKYKELGFQG			
Bovine Trypsinogen	23,965.50	23,965.49	0.01	Six cysteine bridges*
	VDDDDKIVGGYT(C)[*HydrogenLoss*]GANTVPYQVSLNSGYHF(C)[*HydrogenLoss*]GGSLINSQWVVSAAH(C)[*HydrogenLoss*]YKSGIQVRLGEDNINVVEGNEQFISASKSIVHPSYNSNTLNNDIMLIKLKSAASLNSRVASISLPTS(C)[*HydrogenLoss*]ASAGTQ(C)[*HydrogenLoss*]LISGWGNTKSSGTSYPDVLK(C)[*HydrogenLoss*]LKAPILSDSS(C)[*HydrogenLoss*]KSAYPGQITSNMF(C)[*HydrogenLoss*]AGYLEGGKDS(C)[*HydrogenLoss*]QGDSGGPVV(C)[*HydrogenLoss*]SGKLQGIVSWGSG(C)[*HydrogenLoss*]AQKNKPGVYTKV(C)[*HydrogenLoss*]NYVSWIKQTIASN			
Bovine Carbonic Anhydrase	29,005.78	29,006.82	1.04	N-terminal acetylation after the initiator methionine is removed
	[*Acetyl*]SHHWGYGKHNGPEHWHKDFPIANGERQSPVDIDTKAVVQDPALKPLALVYGEATSRRMVNNGHSFNVEYDDSQDKAVLKDGPLTGTYRLVQFHFHWGSSDDQGSEHTVDRKKYAAELHLVHWNTKYGDFGTAAQQPDGLAVVGVFLKVGDANPALQKVLDALDSIKTKGKSTDFPNFDPGSLLPNVLDYWTYPGSLTTPPLLESVTWIVLKEPISVSSQQMLKFRTLNFNAEGEPELLMLANWRPAQPLKNRQVRGFPK			

Cysteine bridge introduces a hydrogen loss on each participating cysteine

Supplementary Table S5. Proteoform features reported by TopFD from the top-down five-protein mixture LC-MS data

**Protein**	**Mass (Dalton)**	**Charge of Seed Envelope**	**Charge Range**	**RT of Seed Envelope (minutes)**	**RT Range (minutes)**
Bovine Ubiquitin	8,559.64	11	6 - 14	16.584	16.07 - 19.18
Bovine Superoxide Dismutase	15,581.83	15	9 - 19	17.811	17.05 - 21.89
Equine Myoglobin	16,941.02	19	9 - 29	22.863	22.49 - 24.07
Bovine Trypsinogen	23,965.50	12	12 - 20	19.367	19.18 - 21.13
Bovine Carbonic Anhydrase	29,005.78	33	21 - 46	23.978	23.60 - 25.97

Supplementary Table S6. Parameter settings for ProMex

**Parameter**	**Value**
Output mass range	Min 600, Max 100,000
Charge range	Min 1, Max 60
Score threshold	-10

Supplementary Table S7. Parameter settings for FlashDeconv

Parameter	Value
Minimum precursor signal noise ratio	1
mzML mass charge	0
Preceding MS1 count	3
Maximum MS level	2
Merging method	0
Tolerance	[10.0, 10.0]
Output mass range	Min 100, Max 100,000
Charge range	Min 1, Max 60
m/*z* range	Min -1, Max -1
RT range	Min -1, Max -1
Minimum isotope cosine	[0.8, 0.8]
Minimum Q-score	0
Minimum peaks	[3, 3]
Minimum intensity	100
RT window	180
Mass error (ppm)	10
Mass error (Da)	-0.1
Quant method	Area
Minimum sample rate	0.2
Minimum trace length	1
Maximum trace length	-1
Minimum isotope cosine	-1

Supplementary Table S8. Parameter settings for Xtract

**Parameter**	**Value**
Source spectrum type	Sliding windows
Use restricted time	Disabled
Chromatogram trace type	TIC
Sensitivity	High
Rel. Intensity threshold (%)	1
Target avg spectrum offset	3
Merge tolerance	30 ppm
Max RT gap	1 minute
Minimum number of detected intervals	1
Deconvolution algorithm	Xtract (isotopically resolved)
Output mass range	Min 100, Max 100,000
Output mass	M
Signal noise ratio threshold	3
Rel. Abundance threshold (%)	0
m/*z* range	Min 400, Max 2000
Charge range	Min 1, Max 60
Minimum number of detected charge states	1
Isotope table	Protein
Calculate XIC	Disabled
Fit factor (%)	80
Remainder threshold (%)	25
Consider overlaps	Enabled
Resolution at 400 m/z	RAW file specific
Negative charge	Disabled
Charge carrier	H+ (1.00727663)
Minimum intensity	1
Expected intensity error	3

Supplementary Table S9. The numbers of all features, valid features, and mass artifacts reported from the OC and SW620 data sets by TopFD, ProMex, FlashDeconv, and Xtract

Data	Tool	# Reported Features	# Valid Features	# Mass Artifacts
Ovarian Cancer – Replicate 1	TopFD	7948	7672	276
	ProMex	12018	5811	6207
	FlashDeconv	6346	6067	279
	Xtract	9319	7773	1546
Ovarian Cancer – Replicate 2	TopFD	8188	7830	358
	ProMex	12043	5819	6224
	FlashDeconv	6133	5832	301
	Xtract	9672	7879	1793
Ovarian Cancer – Replicate 3	TopFD	8445	8146	299
	ProMex	12640	6105	6535
	FlashDeconv	6336	6034	302
	Xtract	9969	8289	1680
Ovarian Cancer – Replicate 4	TopFD	8691	8404	287
	ProMex	13184	6283	6901
	FlashDeconv	6632	6322	310
	Xtract	10324	8672	1652
Ovarian Cancer – Replicate 5	TopFD	8478	8195	283
	ProMex	12694	6164	6530
	FlashDeconv	6442	6131	311
	Xtract	10061	8319	1742
Ovarian Cancer – Replicate 6	TopFD	8290	7985	305
	ProMex	12633	6099	6534
	FlashDeconv	6312	6007	305
	Xtract	10057	8373	1684
Ovarian Cancer – Replicate 7	TopFD	8290	8003	287
	ProMex	12734	6109	6625
	FlashDeconv	6337	6055	282
	Xtract	10388	8609	1779
Ovarian Cancer – Replicate 8	TopFD	8591	8292	299
	ProMex	13166	6440	6726
	FlashDeconv	6464	6129	335
	Xtract	10562	8643	1919
Ovarian Cancer – Replicate 9	TopFD	8591	8255	336
	ProMex	13213	6368	6845
	FlashDeconv	6379	6074	305
	Xtract	10660	8849	1811
Ovarian Cancer – Replicate 10	TopFD	8539	8222	317
	ProMex	13125	6366	6759
	FlashDeconv	6300	6004	296
	Xtract	10852	8931	1921
SW620 colorectal cell line data – Replicate 1	TopFD	14311	11552	2759
	ProMex	6264	3025	3239
	FlashDeconv	15801	12240	3561
	Xtract	10927	8322	2605
SW620 colorectal cell line data – Replicate 1	TopFD	14530	11444	3086
	ProMex	6090	2984	3106
	FlashDeconv	16521	12781	3740
	Xtract	11046	8379	2667
SW620 colorectal cell line data – Replicate 1	TopFD	15528	12113	3415
	ProMex	6320	3136	3184
	FlashDeconv	16663	13034	3629
	Xtract	11968	9101	2867

Supplementary Table S10. The numbers of top valid features kept for comparison of TopFD, ProMex, FlashDeconv, and Xtract in the OC and SW620 replicates

Data	# Valid Features
Ovarian Cancer – Replicate 1	5811
Ovarian Cancer – Replicate 2	5819
Ovarian Cancer – Replicate 3	6034
Ovarian Cancer – Replicate 4	6283
Ovarian Cancer – Replicate 5	6131
Ovarian Cancer – Replicate 6	6007
Ovarian Cancer – Replicate 7	6055
Ovarian Cancer – Replicate 8	6129
Ovarian Cancer – Replicate 9	6074
Ovarian Cancer – Replicate 10	6004
SW620 colorectal cell line data – Replicate 1	3025
SW620 colorectal cell line data – Replicate 1	2984
SW620 colorectal cell line data – Replicate 1	3136

Supplementary Table S11. Eight input attributions of envelope collections in the neural network model for ECScore

**Attribute**	**Description**
EnvCNN Score	EnvCNN score of the aggregate envelope obtained from the envelope collection
Scaled retention time range	Retention time range (in minutes) of the envelope set of the seed envelope scaled by 1/60
Ratio of matched peaks	Ratio between the total number of matched experimental peaks and the total number of theoretical peaks
Total peak intensity with log transformation	Logarithm of the sum of the scaled intensities of all theoretical peaks
Seed charge state	Charge state of the seed envelope
Average correlation of three experimental envelopes	Three experimental envelopes with the seed charge state are extracted from the spectrum of the seed and two neighboring spectra. Pearson's correlation is computed for each pair of the three envelopes and the average correlation of the three pairs is reported.
Scaled charge state range	Charge state range (maximum charge – minimum charge +1) scaled by 1/30
Log ratio for charge state correction	The log ratio for charge state correction is computed using the method in Supplementary Methods S3.3.

References

(1) McCool, E. N.; Xu, T.; Chen, W.; Beller, N. C.; Nolan, S. M.; Hummon, A. B.; Liu, X.; Sun, L. Deep top-down proteomics revealed significant proteoform-level differences between metastatic and nonmetastatic colorectal cancer cells. *Science Advances* **2022**, *8* (51), eabq6348.

(2) Park, J.; Piehowski, P. D.; Wilkins, C.; Zhou, M.; Mendoza, J.; Fujimoto, G. M.; Gibbons, B. C.; Shaw, J. B.; Shen, Y.; Shukla, A. K.; et al. Informed-Proteomics: open-source software package for top-down proteomics. *Nature Methods* **2017**, *14* (9), 909-914. DOI: 10.1038/nmeth.4388.

(3) Ntai, I.; LeDuc, R. D.; Fellers, R. T.; Erdmann-Gilmore, P.; Davies, S. R.; Rumsey, J.; Early, B. P.; Thomas, P. M.; Li, S.; Compton, P. D. Integrated Bottom-Up and Top-Down Proteomics of Patient-Derived Breast Tumor Xenografts. *Molecular & Cellular Proteomics* **2016**, *15* (1), 45-56. DOI: 10.1074/mcp.M114.047480.

(4) Tran, J. C.; Doucette, A. A. Gel-Eluted Liquid Fraction Entrapment Electrophoresis: An Electrophoretic Method for Broad Molecular Weight Range Proteome Separation. *Analytical Chemistry* **2008**, *80* (5), 1568-1573. DOI: 10.1021/ac702197w.

(5) Arauz-Garofalo, G.; Jodar, M.; Vilanova, M.; de la Iglesia Rodriguez, A.; Castillo, J.; Soler-Ventura, A.; Oliva, R.; Vilaseca, M.; Gay, M. Protamine Characterization by Top-Down Proteomics: Boosting Proteoform Identification with DBSCAN. *Proteomes* **2021**, *9* (2), 21.

(6) DeHart, C. J.; Fellers, R. T.; Fornelli, L.; Kelleher, N. L.; Thomas, P. M. Bioinformatics Analysis of Top-Down Mass Spectrometry Data with ProSight Lite. In *Protein Bioinformatics: From Protein Modifications and Networks to Proteomics*, Wu, C. H., Arighi, C. N., Ross, K. E. Eds.; Springer New York, 2017; pp 381-394.

(7) Kessner, D.; Chambers, M.; Burke, R.; Agus, D.; Mallick, P. ProteoWizard: open source software for rapid proteomics tools development. *Bioinformatics* **2008**, *24* (21), 2534-2536. DOI: 10.1093/bioinformatics/btn323.

(8) Liu, X.; Inbar, Y.; Dorrestein, P. C.; Wynne, C.; Edwards, N.; Souda, P.; Whitelegge, J. P.; Bafna, V.; Pevzner, P. A. Deconvolution and Database Search of Complex Tandem Mass Spectra of Intact Proteins. *Molecular & Cellular Proteomics* **2010**, *9* (12), 2772-2782. DOI: 10.1074/mcp.M110.002766 (acccessed 2021/04/27).

(9) Kou, Q.; Wu, S.; Liu, X. A new scoring function for top-down spectral deconvolution. *BMC Genomics* **2014**, *15* (1), 1-10. DOI: 10.1186/1471-2164-15-1140.

(10) Senko, M. W.; Beu, S. C.; McLaffertycor, F. W. Determination of monoisotopic masses and ion populations for large biomolecules from resolved isotopic distributions. *Journal of the American Society for Mass Spectrometry* **1995**, *6* (4), 229-233. DOI: 10.1016/1044-0305(95)00017-8.

(11) Horn, D. M.; Zubarev, R. A.; McLafferty, F. W. Automated reduction and interpretation of high resolution electrospray mass spectra of large molecules. *Journal of the American Society for Mass Spectrometry* **2000**, *11* (4), 320-332. DOI: 10.1016/S1044-0305(99)00157-9.

(12) Kingma, D. P.; Ba, J. Adam: A method for stochastic optimization. *arXiv preprint arXiv:1412.6980* **2014**.

(13) Bellew, M.; Coram, M.; Fitzgibbon, M.; Igra, M.; Randolph, T.; Wang, P.; May, D.; Eng, J.; Fang, R.; Lin, C. A suite of algorithms for the comprehensive analysis of complex protein mixtures using high-resolution LC-MS. *Bioinformatics* **2006**, *22* (15), 1902-1909. DOI: 10.1093/bioinformatics/btl276.

(14) Tautenhahn, R.; Böttcher, C.; Neumann, S. Highly sensitive feature detection for high resolution LC/MS. *BMC bioinformatics* **2008**, *9* (1), 1-16. DOI: 10.1186/1471-2105-9-504.

(15) Cox, J.; Mann, M. MaxQuant enables high peptide identification rates, individualized ppb-range mass accuracies and proteome-wide protein quantification. *Nature biotechnology* **2008**, *26* (12), 1367-1372. DOI: 10.1038/nbt.1511.

(16) Teleman, J.; Chawade, A.; Sandin, M.; Levander, F.; Malmström, J. Dinosaur: a refined open-source peptide MS feature detector. *Journal of proteome research* **2016**, *15* (7), 2143-2151. DOI: 10.1021/acs.jproteome.6b00016

(17) Zohora, F. T.; Rahman, M. Z.; Tran, N. H.; Xin, L.; Shan, B.; Li, M. DeepIso: a deep learning model for peptide feature detection from LC-MS map. *Scientific reports* **2019**, *9* (1), 1-13. DOI: 10.1038/s41598-019-52954-4.

(18) Zeng, X.; Ma, B. MSTracer: A Machine Learning Software Tool for Peptide Feature Detection from Liquid Chromatography–Mass Spectrometry Data. *Journal of Proteome Research* **2021**, *20* (7), 3455-3462. DOI: 10.1021/acs.jproteome.0c01029
